# Molecular Dynamics Study of CO_2_-Induced Transfer of Crude Oil Components: Roles of Molecular Structure, Cohesion, and Mixture Composition

**DOI:** 10.3390/molecules31183140

**Published:** 2026-09-08

**Authors:** Jiahao Gao, Mingyuan Wang, Yu Zhang, Weifeng Lyu, Ke Zhang, Ziyang Zuo

**Affiliations:** 1 Research Institute of Petroleum Exploration & Development, China National Petroleum Corporation, Beijing 100083, China; gaoplus0108@126.com (J.G.);; 2 State Key Laboratory of Enhanced Oil & Gas Recovery, China National Petroleum Corporation, Beijing 100083, China; 3 University of Chinese Academy of Sciences, Beijing 100049, China

**Keywords:** molecular dynamics, solubility parameter, extraction characteristics, supercritical CO_2_, crude oil components, intermolecular interaction

## Abstract

Molecular dynamics simulations examined the roles of molecular structure, thermodynamic compatibility, intermolecular association, and mixture composition in the supercritical CO_2_ extraction of ten crude oil components at 363.15 K and 15 MPa. Single-component extraction ratios ranged from 80.70% for n-hexane to 5.85% for 2-naphthol. Compounds of similar size differed widely, indicating that topology, aromaticity, and polar functional groups were more informative than molecular size alone. CO_2_ solubility parameters obtained from MD agreed with estimates derived from NIST data, and *δ_CO_*_2_ = 7.28*ρ_r_* captured their reduced density dependence over 303.15–363.15 K. Extraction generally decreased with increasing oil–CO_2_ solubility parameter difference. In binary systems evaluated using oil boundaries determined by the half density criterion, higher fractions of nonpolar partners were associated with increased total extraction ratios, whereas higher fractions of polar partners were associated with decreases. Multicomponent systems showed redistribution that depended on the overall composition, and relative diffusion coefficients qualitatively reflected mobility differences. The gas–oil interaction competition factor, *R_comp_*, decreased from 1.985 to 0.087 in the same order as the extraction ratios. Both the thermodynamic and energetic correspondences persisted after excluding 2-naphthol. Configurations and radial distribution functions showed that association in nonpolar hydrocarbons was dominated by dispersion interactions, whereas polar and aromatic components exhibited additional hydrogen bonding, aromatic stacking, and dipolar or electrostatic organization. Local CO_2_ enrichment near polar sites alone did not explain overall extraction. Overall, the selective transfer of individual components was consistent with a balance between local CO_2_–oil association and collective oil–oil cohesion that depended on mixture composition, while molecular organization may regulate the accessibility of favorable CO_2_ contact sites.

## 1. Introduction

CO_2_-enhanced oil recovery (CO_2_-EOR) has received sustained attention because it can improve hydrocarbon production while offering opportunities for the utilization and associated geological storage of anthropogenic CO_2_ [1,2]. Under reservoir conditions, dissolved CO_2_ can induce oil swelling, viscosity reduction, interfacial-tension reduction, and changes in phase behavior, thereby improving oil mobility and displacement efficiency [3,4]. In addition to these bulk-fluid effects, particular oil components may transfer from the oil-rich phase into the CO_2_-rich phase under high-pressure or near-miscible conditions [5,6]. Here, component extraction refers to the transfer of oil molecules from an oil-rich domain into a CO_2_-rich environment. This process is important because crude oil contains molecules with different carbon numbers, molecular architectures, and functional groups. Unequal partitioning of these components may progressively alter mass transfer and fluid properties, thereby affecting mass transfer and displacement behavior [7]. Consequently, overall recovery measurements alone cannot fully describe why chemically distinct oil molecules exhibit different transfer tendencies. A component-resolved understanding of CO_2_ extraction is therefore needed to clarify how molecular diversity influences compositional mass transfer and to connect molecular-scale behavior with the macroscopic performance of CO_2_ flooding [8].

Current understanding of CO_2_–oil interactions has been developed through experimental, thermodynamic, and molecular-simulation approaches. High-pressure phase-equilibrium measurements, swelling tests, interfacial-tension measurements, and compositional analyses provide direct evidence of bulk-fluid responses and phase-composition changes under reservoir-relevant conditions [9,10,11,12]. Such experiments are essential for evaluating macroscopic displacement mechanisms, although their outputs are generally phase-averaged and provide limited resolution of the molecular events underlying component-selective transfer. Thermodynamic models, particularly cubic equations of state like the Soave–Redlich–Kwong and Peng–Robinson equations coupled with fluid characterization, mixing rules, and binary interaction parameters, are widely used to predict phase equilibria, CO_2_ solubility, minimum miscibility pressure, and compositional redistribution over broad temperature and pressure ranges [13,14,15]. Cohesive-energy-based descriptors, including Hildebrand and Hansen solubility parameters, further offer a compact representation of the thermodynamic compatibility between CO_2_ and different oil constituents [16,17]. Their applicability to complex oils depends on parameter accuracy and molecular representation. Molecular simulations, particularly molecular dynamics simulations, provide complementary access to diffusion, intermolecular energies, molecular association, and the local distribution of CO_2_ around molecular sites [18]. Their interpretation nevertheless depends on force-field quality, model composition, accessible length and time scales, and statistical sampling. These approaches provide a basis for describing CO_2_–oil phase behavior and transport, but their different levels of description must be connected to understand component-selective extraction.

Despite these advances, several aspects of component-selective CO_2_ extraction remain insufficiently understood. Molecular simulations have clarified the behavior of individual hydrocarbons and selected polar or aromatic compounds, but systematic comparisons among chemically distinct molecular classes under consistent thermodynamic and modeling conditions have received comparatively limited attention [19]. Previous molecular simulations have generally focused on hydrocarbons with a single structure or on oil components whose molecular structure and carbon number vary simultaneously. For example, studies have examined the interactions between CO_2_ and saturated hydrocarbons, aromatic hydrocarbons, resins, or asphaltene molecules, and have highlighted the competitive relationship between gas–oil interactions and oil–oil association [8,20,21]. Although the effects of different molecular structures and carbon numbers have been investigated, systematic studies that isolate molecular-structure effects at a fixed carbon number remain limited. These studies have provided important molecular-scale insights into CO_2_–oil interactions and mass transfer, but comparatively limited attention has been given to controlled cross-class comparisons in which molecular size, topology, aromaticity, and polar functionality are systematically varied under consistent thermodynamic and simulation conditions. Consequently, the extent to which descriptors such as carbon number, molecular mass, molecular architecture, or functionality can be transferred across different classes of oil components remains uncertain. Similarly, thermodynamic quantities such as solubility-parameter differences characterize bulk compatibility, whereas their connections with molecular mobility, self-association, and the accessibility of specific CO_2_-contact sites have not been fully established [20]. For multicomponent oils, existing studies have commonly emphasized total dissolution, average transport properties, or overall phase behavior. Composition-dependent coupling has received less systematic consideration [22]. It therefore remains unclear how the identity and proportion of coexisting components modify component transfer within binary and multicomponent oils. The central scientific question is whether component-selective extraction can be explained by molecular structure and thermodynamic compatibility, or whether component-dependent oil–oil association and CO_2_–oil interactions must also be considered.

To address these questions, a molecular model comprising a spherical oil droplet surrounded by CO_2_ was constructed to represent the mass transfer of oil molecules from an oil-rich domain into a CO_2_-rich environment. Representative components with different carbon numbers, molecular architectures, and functional groups were considered under consistent conditions. Pure-component systems were used to examine the relationships between molecular characteristics and intrinsic extraction behavior, whereas binary mixtures with varying compositions were introduced to evaluate mutual component influences and compositional dependence. Multicomponent oil models were further employed to examine whether knowledge derived from pure components remained applicable to chemically complex mixtures. Solubility parameters derived from cohesive energy were evaluated to assess whether thermodynamic compatibility could provide a useful description of component-dependent extraction. Oil–oil and CO_2_–oil interaction energies, representative molecular configurations, and radial distribution functions were combined to examine molecular association and local CO_2_ distributions. This multilevel strategy was intended to relate thermodynamic compatibility and mass transfer to the intermolecular organization of both pure and mixed oil systems.

Accordingly, the principal contribution of this study lies in systematically distinguishing molecular-size effects from differences associated with molecular structure and functionality, to extend the analysis from single-component behavior to composition-dependent coupling in binary and multicomponent systems, and to integrate thermodynamic compatibility with molecular mobility, intermolecular energetics, and local structural organization. In particular, the comparison among components with the same or similar molecular size provides an assessment of the of molecular topology and chemical functionality beyond conventional size-based descriptors. The solubility-parameter difference further provides a thermodynamic descriptor for comparing component-specific transfer tendencies, while the combined energetic and structural analyses provide a multilevel basis for interpreting the competition between CO_2_–oil interaction and oil–oil cohesion.

## 2. Results and Discussion

### 2.1. Association of Component Type and Molecular Structure with CO_2_ Extraction Ratio

**Figure 1:**
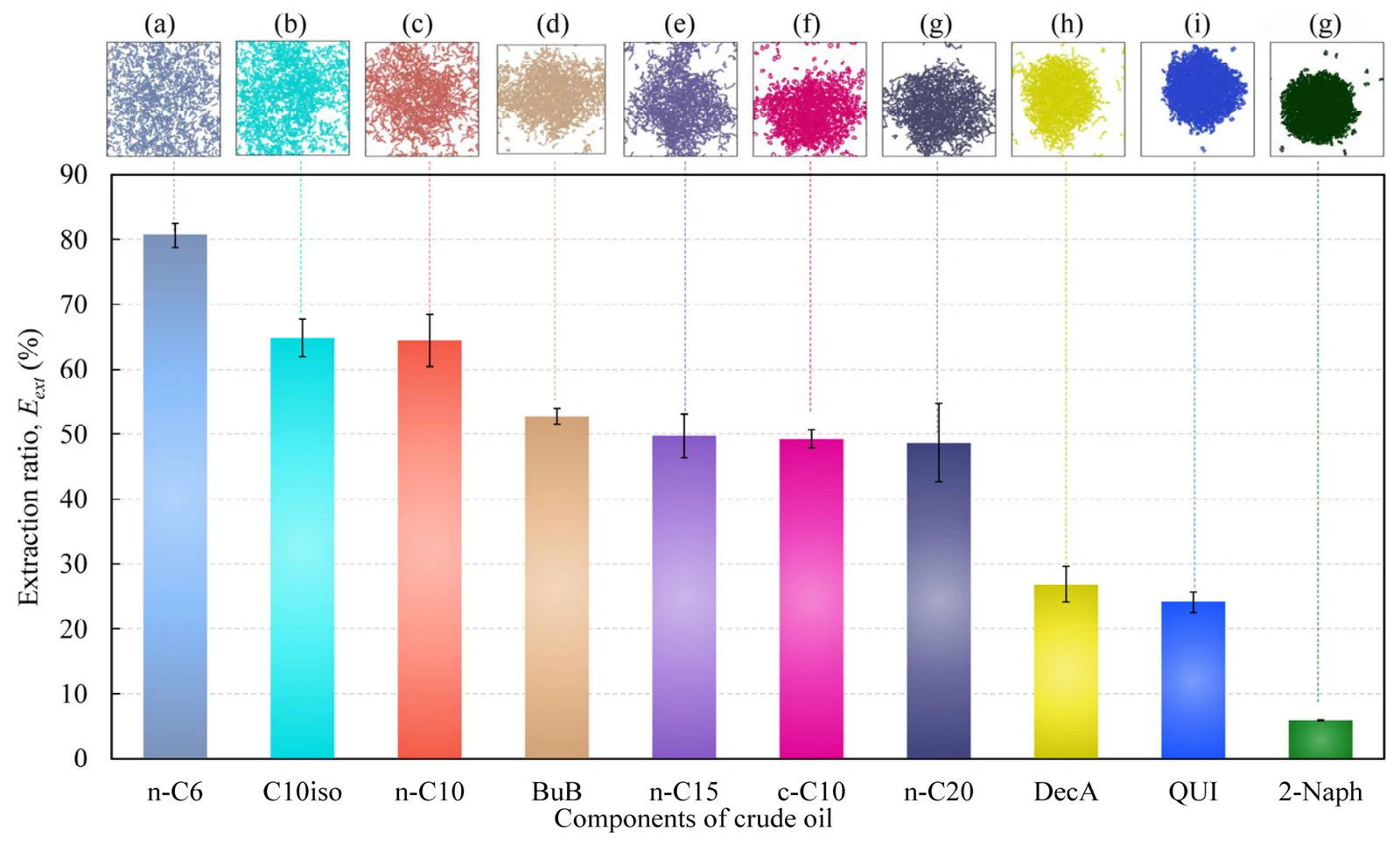
Molecular configurations and extraction ratios of ten pure crude-oil components in supercritical CO_2_ at 363.15 K and 15 MPa. The configurations were obtained at 800 ps, and the extraction ratios were averaged over the stable period from 800 to 1000 ps.

The investigated components of crude oil exhibited strongly differentiated CO_2_ extraction characteristics at 363.15 K and 15 MPa. The extraction ratio *E_ext_* ranged from 5.85% for 2-Naph to 80.70% for n-C6, corresponding to an overall difference of 74.85% (Figure 1). The highest values were obtained for n-C6, C10iso, and n-C10 at 80.70%, 64.82%, and 64.43%, respectively, whereas BuB, n-C15, c-C10, and n-C20 exhibited intermediate extraction ratios ranging from 48.63 to 52.73%. By comparison, DecA, QUI, and 2-Naph showed substantially lower values of 26.87%, 24.16%, and 5.85%, respectively. The configurations recorded at stability provide a visualization of the molecular distributions underlying these calculated extraction ratios, with different components retaining markedly different proportions of molecules within the central oil-rich region. The boundary-radius sensitivity analysis (Figure S1) showed that changing the operational radius from 37 to 43 Å affected the absolute extraction values but did not alter the principal component-dependent trends and ranking. The extraction ratios obtained from the three independent simulations are summarized in Table S1 of the Supplementary Information.

**Figure 2:**
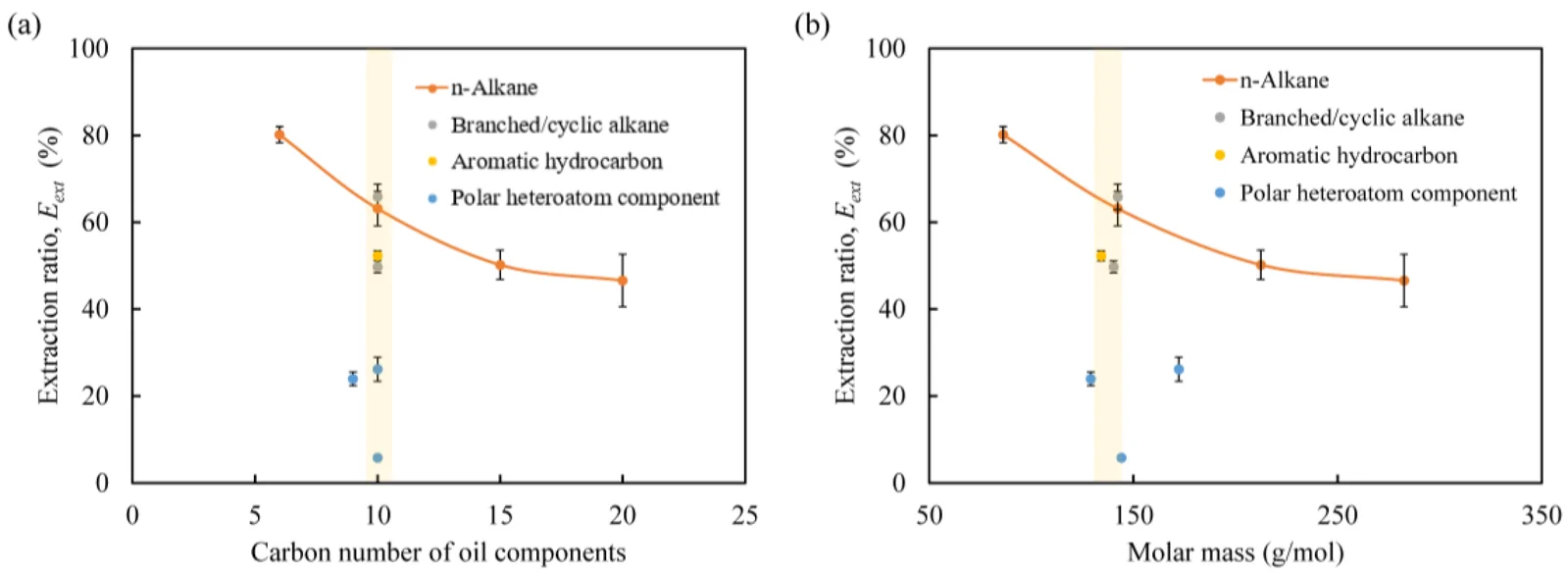
Relationships between molecular size descriptors and the extraction ratios of pure crude-oil components: (**a**) carbon number and (**b**) molecular mass.

An apparent molecular-size dependence emerged when the comparison was restricted to the consistent n-alkane homologous series. The extraction ratio decreased monotonically from n-C6 to n-C10, n-C15, and n-C20 (Figure 2). Increasing the carbon number from 6 to 20 was accompanied by a reduction of 32.07%. However, the magnitude of this reduction was not uniform: the decrease was 16.27% from n-C6 to n-C10 but only 1.08% from n-C15 to n-C20. Because carbon number, molar mass, and chain length covary within this homologous series, their individual contributions cannot be separated from the present dataset. The observed trend reflects the combined influence of increased molecular size, reduced diffusion coefficient, and stronger intermolecular dispersion with increasing chain length [18]. Accordingly, carbon number and molar mass describe a consistent trend within the n-alkane series.

The size-related trend observed for n-alkanes did not extend uniformly across chemically distinct components. The six components with ten carbons exhibited extraction ratios ranging from 5.85% for 2-Naph to 64.82% for C10iso, producing a difference of 58.97% despite their identical carbon number (Figure 2a). A comparable divergence was obtained among components with similar molar masses: C10iso, n-C10, c-C10, BuB, and 2-Naph all fell within approximately 134–144 g/mol, while their extraction ratios again spanned approximately 60% (Figure 2b). The absence of a simple mass dependence is further illustrated by n-C20, which had the highest molar mass of 282.55 g mol^−1^ but retained an extraction ratio of 48.63%. This value exceeded those of DecA and 2-Naph by 21.76% and 42.78%, respectively, although their molar masses were only 172.26 and 144.164 g mol^−1^. These counterexamples demonstrate that neither carbon number nor molar mass alone consistently differentiates the extraction responses of components belonging to different chemical classes.

The marked variation among components of comparable size supports the need to consider the molecular architecture and chemical functionality when interpreting their extraction behavior. Among the hydrocarbons with ten carbons, the C10iso and n-C10 exhibited extraction ratios of 64.82% and 64.43%, whereas BuB and c-C10 gave lower values of 52.73% and 49.25%, respectively. The 15.18% difference between n-C10 and c-C10 indicates that open-chain and cyclic molecular frameworks were associated with different extraction characteristics under the present conditions. Cyclic and aromatic frameworks may modify conformational freedom, molecular packing, and molecular mobility, thereby altering the probability of oil molecules leaving the oil phase. Nevertheless, the difference between C10iso and n-C10 was only 0.39%, and the present results therefore do not independently establish a general enhancement arising from molecular branching.

The low extraction ratios of the heteroatom containing components further indicate that their behavior cannot be interpreted solely from carbon number, molar mass, or polarity. DecA and QUI exhibited extraction ratios of 26.87% and 24.16%, respectively, whereas the value for 2-Naph was only 5.85%. Heteroatom functionality affects both oil–oil association and interaction with CO_2_, while aromaticity and functional group identity introduce additional structural differences [23]. Because these characteristics vary simultaneously among DecA, QUI, and 2-Naph, their individual contributions cannot be isolated from the present comparison.

To examine whether the low extraction of 2-Naph was associated with crystalline ordering, intermolecular center of mass RDFs were calculated within central spherical regions of 15, 20, and 25 Å (Figure S2). All three profiles exhibited a broad first coordination feature at approximately 6–7 Å, followed by a weak second shell that gradually decayed toward unity. Persistent narrow peaks over successive coordination shells were not observed, and the principal features remained consistent among the three core radii. The simulated 2-Naph rich domain therefore showed noncrystalline, disordered translational organization with liquid-like short-range correlations. The RDF analysis does not uniquely distinguish an amorphous state from a metastable liquid or a CO_2_ plasticized state. Accordingly, this structural description is limited to the finite CO_2_ contacted domain sampled under the present molecular model and simulation conditions and is not interpreted as a direct prediction of the equilibrium bulk phase of pure 2-Naph. The observed extraction differences should therefore not be attributed to polarity or equilibrium phase state alone.

### 2.2. Thermodynamic Description of CO_2_ Extraction Characteristics Based on Solubility Parameter

The solubility parameter *δ* provides a thermodynamic description of fluids in molecular cohesion, due to the definition of Hildebrand parameter as the square root of the cohesive energy density (*CED*) [16], as shown in Equation (1):

(1) $$\delta ={\left(CED\right)}^{1/2}={\left(\frac{E_{coh}}{V}\right)}^{1/2}$$

where *E_coh_* and *V* denote the molar cohesive energy and volume, respectively. The Hansen solubility parameter *δ_Hansen_* is expressed as Equation (2), and relative values were obtained from the cited source [17]:

(2) $$\delta_{Hansen}={\left({\delta_{D}}^{2}+{\delta_{P}}^{2}+{\delta_{H}}^{2}\right)}^{1/2}$$

where *δ_D_*, *δ_P_*, and *δ_H_* represent the dispersion, polar, and hydrogen-bonding contributions, respectively. The MD-derived values of the investigated oil components showed a component ranking highly consistent with that obtained from the Hansen parameters with a mean relative deviation of 3.99% in Table 1. Thus, the MD correctly captured relative differences in cohesive thermodynamic properties among oil components with different carbon numbers, molecular frameworks, and functional groups.

**Figure 3:**
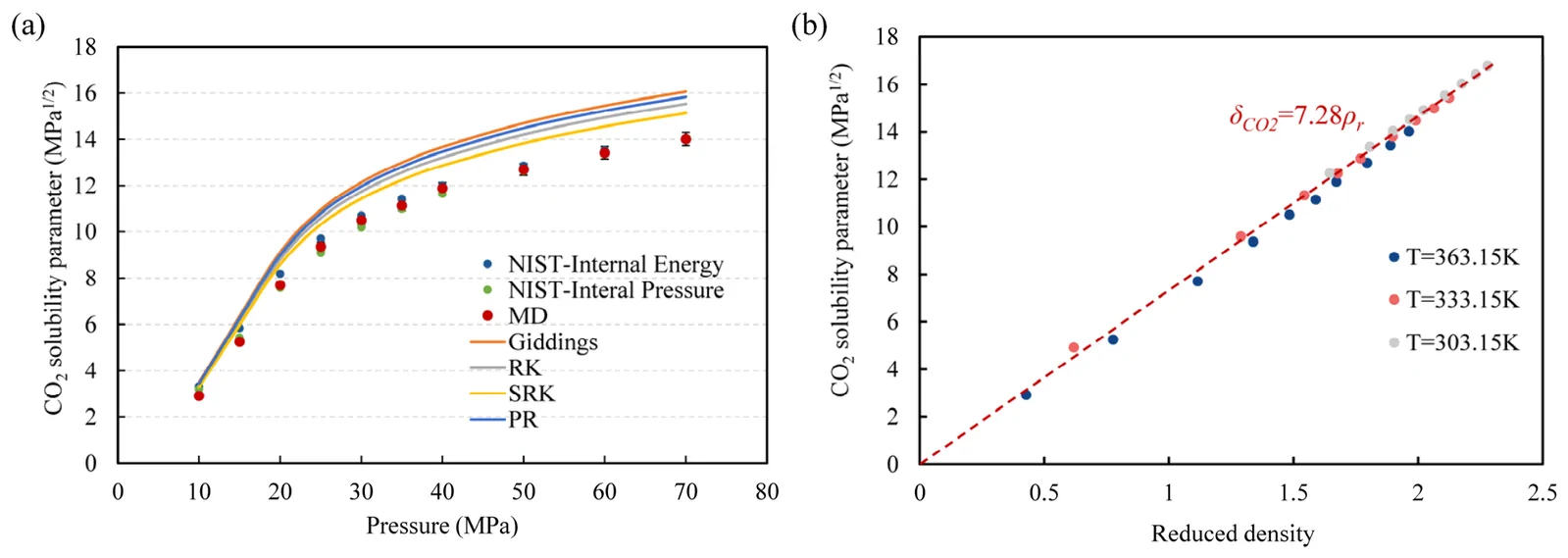
Solubility parameter of supercritical CO_2_: (**a**) comparison of the MD results with values derived from the NIST internal-energy and internal-pressure methods and with predictions from the Giddings, RK, SRK, and PR equations of state at 363.15 K; (**b**) relationship between the MD-derived CO_2_ solubility parameter and reduced density at 303.15–363.15 K.

The reliability of the MD procedure for *δ* of supercritical CO_2_ was evaluated at 363.15 K over 10–70 MPa using two reference datasets derived from NIST thermophysical properties. The internal-energy and internal-pressure methods from Equation (3) were applied as [24]:

(3) $$\delta_{U}={\left[\frac{{U_{m}}^{ig}-U_{m}}{V_{m}}\right]}^{1/2}={\left(-\frac{{U_{m}}^{R}}{V_{m}}\right)}^{1/2}$$

and

(4) $$\delta_{P}={\left[T{\left(\frac{\partial P}{\partial T}\right)}_{V}-P\right]}^{1/2}$$

respectively, where *U_m_^ig^*, *U_m_*, and *U_m_^R^* are the ideal-gas, real-fluid, and residual molar internal energies, respectively. For comparison, the *δ* of supercritical CO_2_ based on the Giddings correlation and the RK, SRK, and PR equations of state (EOS) were evaluated using their published formulations and parameter sets [25,26]. As shown in Figure 3a, the MD results reproduced the nonlinear increase in the CO_2_ solubility parameter with pressure and remained between the two NIST-derived reference values over 10–70 MPa. The MD derived values were closer to the reference values than the predictions obtained from the four selected equation of state models. At 70 MPa, the MD value of 14.014 MPa^1/2^ differed from the values derived using the internal energy and internal pressure methods by only 0.045 and 0.052 MPa^1/2^, respectively. Among the four equation of state models, SRK produced the smallest deviations, although its predictions remained less consistent with the reference estimates than the MD results. Overall, the MD derived values showed reasonable agreement with the two reference datasets over the investigated state points.

Reduced density *ρ_r_* was subsequently introduced to establish a correlation that integrates the effects of temperature and pressure:

(5) $$\rho_{r}=\frac{\rho }{\rho_{c}}$$

where *ρ* is the CO_2_ density at the specified temperature and pressure, and *ρ_c_* is the critical density of 0.469 g/cm^3^. At each investigated temperature and pressure, the CO_2_ solubility parameter increased approximately linearly with *ρ_r_* as shown in Figure 3b. Table 2 indicated the temperature-specific correlations yielded *R*^2^ (coefficient of determination) values above 0.997. A zero-intercept fit to all 28 data points produced the unified empirical correlation:

(6) $$\delta_{CO_{2}}=k_{CO_{2}}\rho_{r}$$

where *k_CO_*_2_ = 7.28MPa^1/2^. Within 303.15–363.15 K and *ρ_r_* of 0.427–2.279, the correlation yielded *R*^2^ = 0.995 and a MAPE of 2.35%. The individual slopes *k_T_* at 363.15, 333.15, and 303.15 K were 7.064, 7.299, and 7.384 MPa^1/2^, respectively, corresponding to a maximum variation of 4.5%. These results indicate that reduced density captures most of the combined temperature and pressure dependence within the investigated dataset. Nevertheless, the variation among the temperature specific slopes indicates that a residual temperature dependence remains. This residual contribution is not represented explicitly in the simplified empirical correlation of Equation (6), which should therefore be applied only within the investigated temperature and reduced density ranges.

The thermodynamic correspondence between the oil and gas phases was quantified using the absolute difference in solubility parameters as shown in Equation (7).

(7) $$\Delta \delta =\left|\delta_{oil}-\delta_{CO_{2}}\right|$$

**Figure 4:**
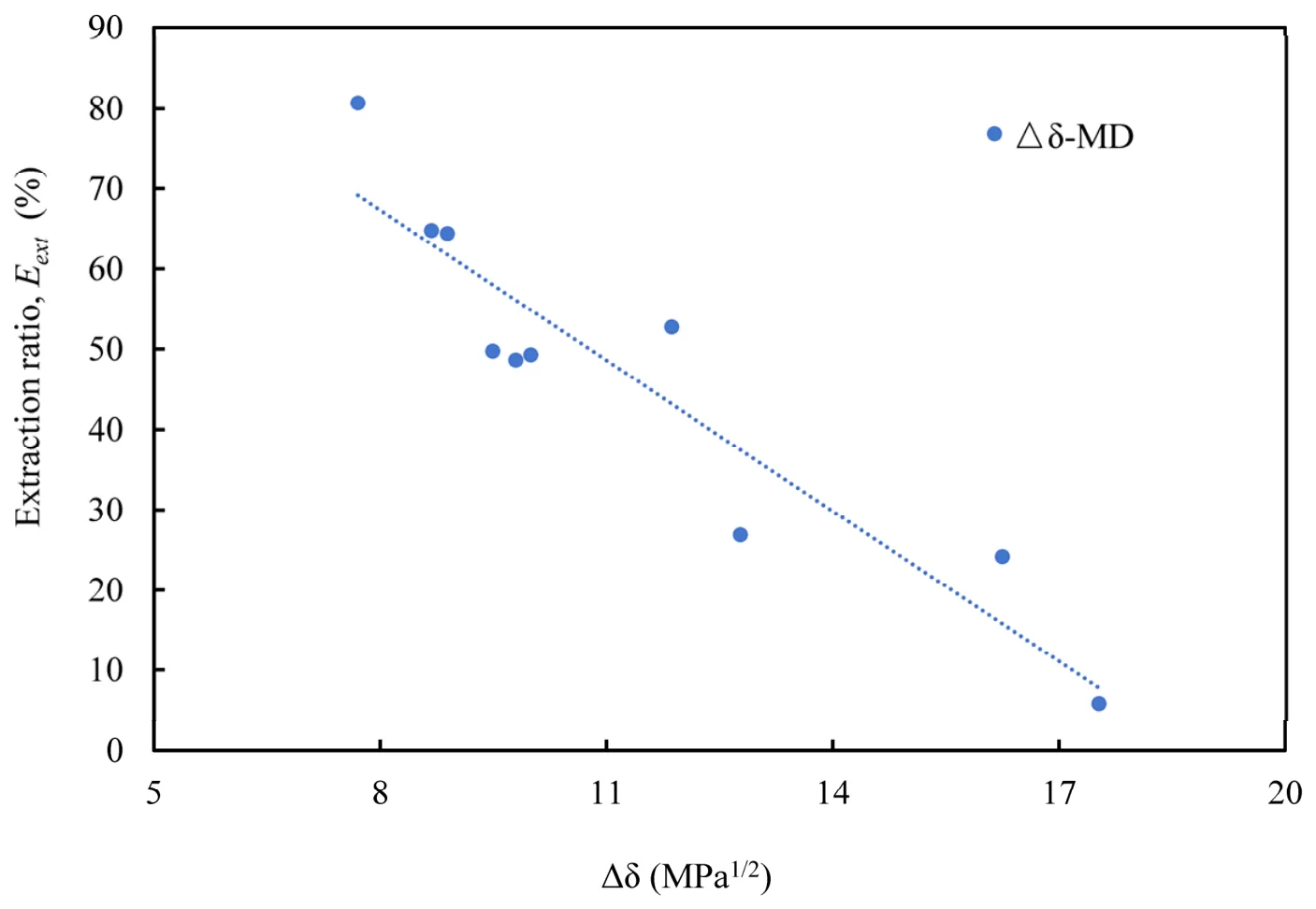
Relationship between the MD-derived oil–CO_2_ solubility-parameter difference and the extraction characteristics of the pure crude-oil components at 363.15 K and 15 MPa.

At 363.15 K and 15 MPa, *δ_CO_*_2_ was 5.728 MPa^1/2^. The corresponding relationship between extraction ratio of oil components and Δ*δ* was shown in Figure 4. As Δ*δ_MD_* increased from 7.700 MPa^1/2^ for n-C6 to 17.515 MPa^1/2^ for 2-Naph, the extraction ratio decreased from 80.70% to 5.85%. A descriptive negative linear relationship was obtained over the investigated component set, as expressed in Equation (8).

(8) $$E_{ext}=119.80-6.24\Delta \delta_{MD}$$

A smaller Δ*δ* may indicate a closer overall match in the types and strengths of intermolecular interactions represented by the solubility parameter. Such matching could reduce the thermodynamic penalty associated with transferring oil molecules from the oil phase into the CO_2_-rich phase. This interpretation will be examined further through the interaction analysis in Section 2.4. Accordingly, Δ*δ* provides a potential thermodynamic approach for describing and comparing the extraction capability of CO_2_ toward oil components with different carbon numbers, molecular structures, and polar functionalities. Nevertheless, the relationship was not strictly mathematical correspondence. BuB exhibited a larger Δ*δ_MD_* than c-C10, at 11.858 and 9.988 MPa^1/2^, respectively, but its extraction ratio was slightly higher, at 52.73% compared with 49.25%. DecA and QUI also exhibited relatively similar extraction ratios of 26.87% and 24.16%, despite their Δ*δ_MD_* values differing by 3.471 MPa^1/2^. These deviations suggest that molecular aggregation, mobility, and specific oil–CO_2_ interactions may contribute additional effects that are not fully represented by a single total solubility-parameter difference. Overall, solubility parameters and the resulting oil–CO_2_ Δ*δ* provide a quantitative thermodynamic framework for comparing the extraction characteristics of structurally diverse crude-oil components.

The negative correspondence between Δ*δ_MD_* and extraction ratio was retained after excluding 2-Naph from the analysis. The Pearson coefficient changed from −0.933 for all ten components to −0.882 for the remaining nine components, while the Spearman coefficient changed from −0.915 to −0.883 (Table S2). Thus, although 2-Naph increased the apparent strength of the relationship to some extent, the principal thermodynamic correspondence was not dependent on this extreme data point.

### 2.3. Extraction Behavior of Binary and Multicomponent Oil Systems

**Figure 5:**
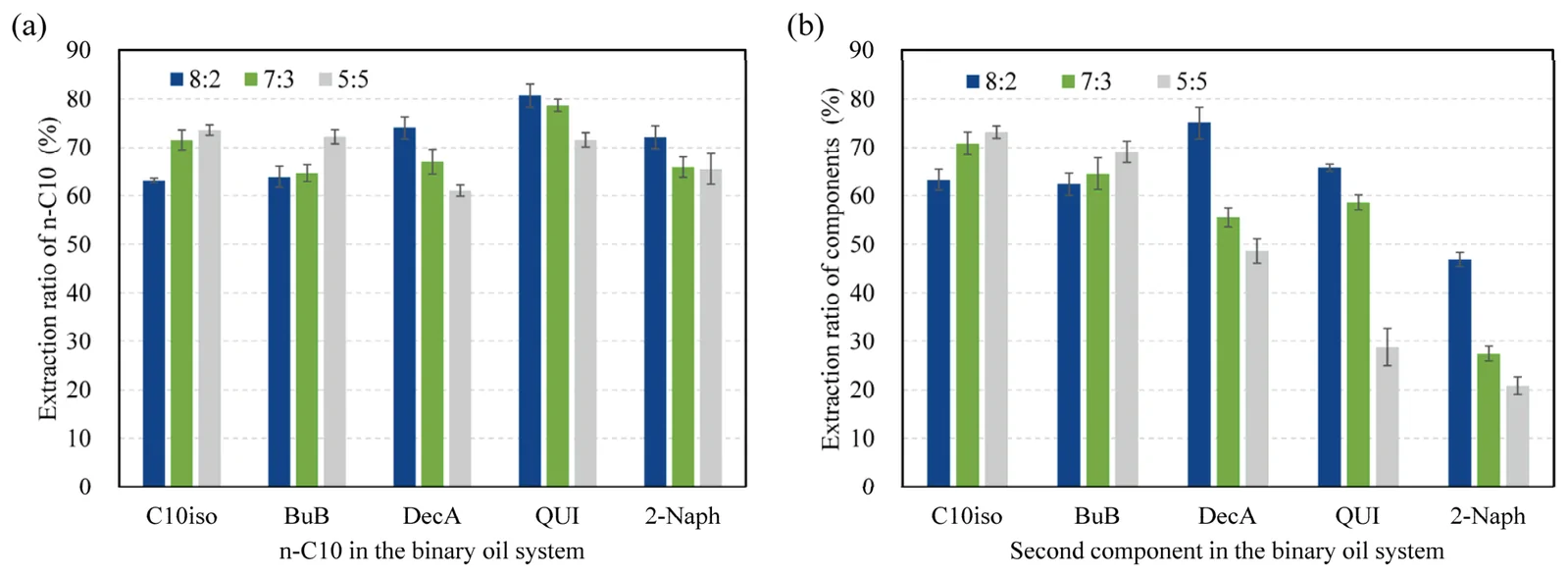

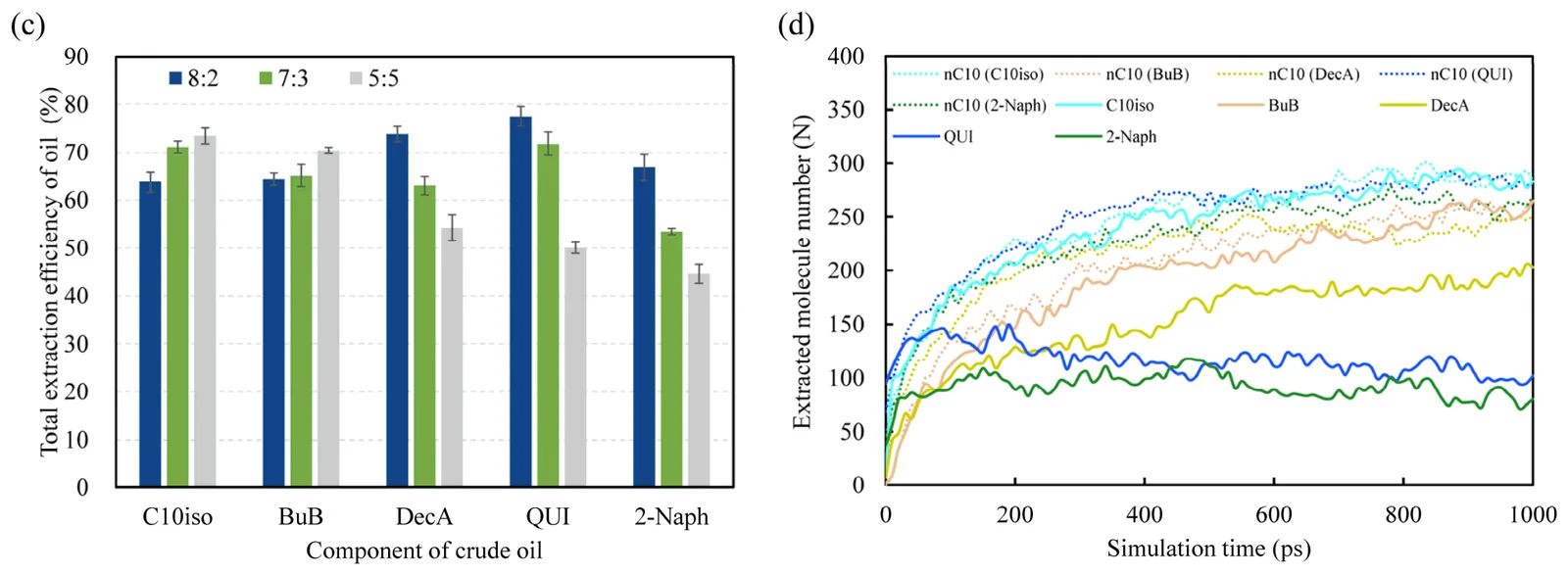
Extraction characteristics of binary oil systems at n-C10 to second component molecular ratios of 8:2, 7:3, and 5:5: (**a**) extraction ratio of n-C10; (**b**) extraction ratio of the second component; (**c**) total extraction ratio of the binary oil system; and (**d**) temporal evolution of the numbers of extracted n-C10 and second component molecules in the 5:5 systems.

The extraction behavior of the binary oil systems varied with both the identity and proportion of the second component. For each mixed system, the extraction boundary was determined using the half density criterion, and the resulting extraction ratios and boundary positions are reported in Tables S3 and S4 in the Supplementary Information, respectively. Because the mixed and single-component systems differ in model construction and boundary determination, their extraction ratios are not compared directly. As shown in Figure 5a–c, increasing the fraction of C10iso or BuB was accompanied by higher extraction ratios for both n-C10 and the second component, leading to an increase in the total extraction ratio. By contrast, the extraction ratios of both components generally decreased as the fractions of DecA, QUI, and 2-Naph increased, with a corresponding decline in total extraction. These contrasting trends indicate that the response of a binary system to composition changes depends on the identity of the second component.

The temporal evolution of the extracted molecule numbers provides further evidence that the overall extraction response arose from different transfer behaviors of the two components. At the 5:5 composition, the extracted molecule numbers of n-C10 and C10iso or BuB followed broadly similar trajectories and approached comparable levels during the later simulation period (Figure 5d), consistent with the relatively small differences between their component extraction ratios in Figure 5a,b. In the n-C10/DecA system, the extracted molecule number of DecA remained lower than that of n-C10 after the initial stage, resulting in a more distinct separation between the two components. This difference was more pronounced in the systems containing QUI and 2-Naph, for which the extracted molecule numbers of the second components remained considerably below those of n-C10 during the later stage. These differences observed over time are consistent with the component extraction ratios at the 5:5 composition and help explain the corresponding differences in total extraction shown in Figure 5c. The total extraction response therefore reflects the extent to which the second component can be transferred together with n-C10 under a given composition.

Relative molecular mobility provides complementary evidence for the component dependent extraction observed in the binary systems. Because the boundaries determined using the half density criterion were generally smaller than 40 Å, the diffusion coefficients calculated for oil molecules beyond 40 Å were derived, allowing the mobility comparison to focus on molecules that had entered the CO_2_ rich region. The mobility of each second component relative to n-C10 in the same system was expressed as

(9) $$R_{D,i}=\frac{D_{i}}{D_{n-C10}}$$

**Figure 6:**
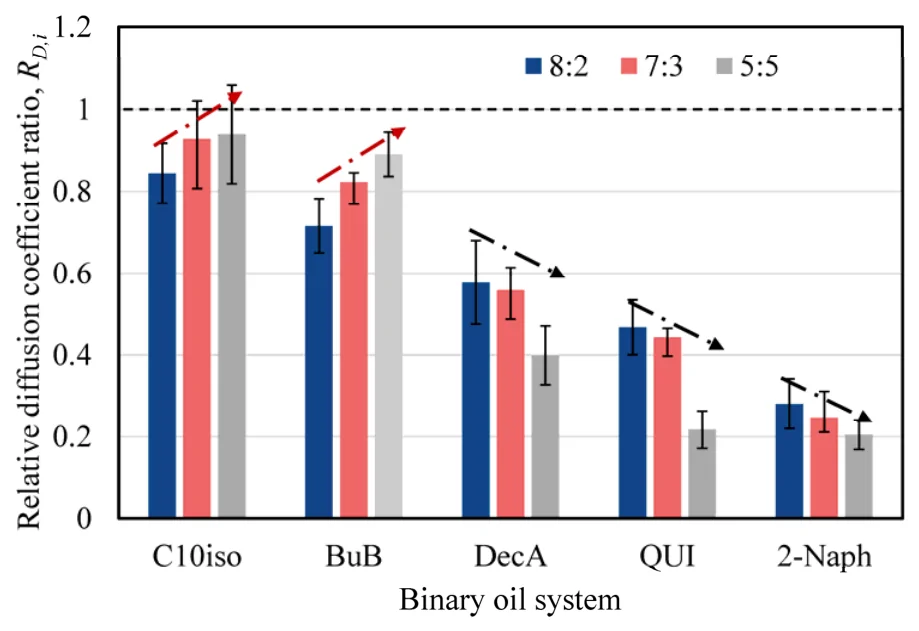
Relative diffusion-coefficient ratios of the second oil components to n-C10 in the CO_2_ region for binary oil systems at different n-C10-to-second-component molecular ratios.

As shown in Figure 6, all *R_D_*_,*i*_ values were below unity. When the fraction of the second component increased, the ratios for C10iso and BuB increased from 0.844 to 0.939 and from 0.716 to 0.890, respectively. DecA showed little change between the 8:2 and 7:3 compositions before decreasing to 0.399 at 5:5, whereas the ratios for QUI and 2-Naph progressively decreased to 0.227 and 0.203. These variations broadly correspond to the extraction characteristics in Figure 5, with the increasing relative mobility of C10iso and BuB accompanying their enhanced extraction at higher fractions, while the lower relative mobilities of DecA, QUI, and 2-Naph at 5:5 were associated with greater differences from n-C10. Nevertheless, the correspondence was not strictly quantitative, indicating that the relative diffusion coefficient is more appropriately interpreted as a qualitative measure of molecular mobility rather than an independent predictor of extraction.

**Figure 7:**
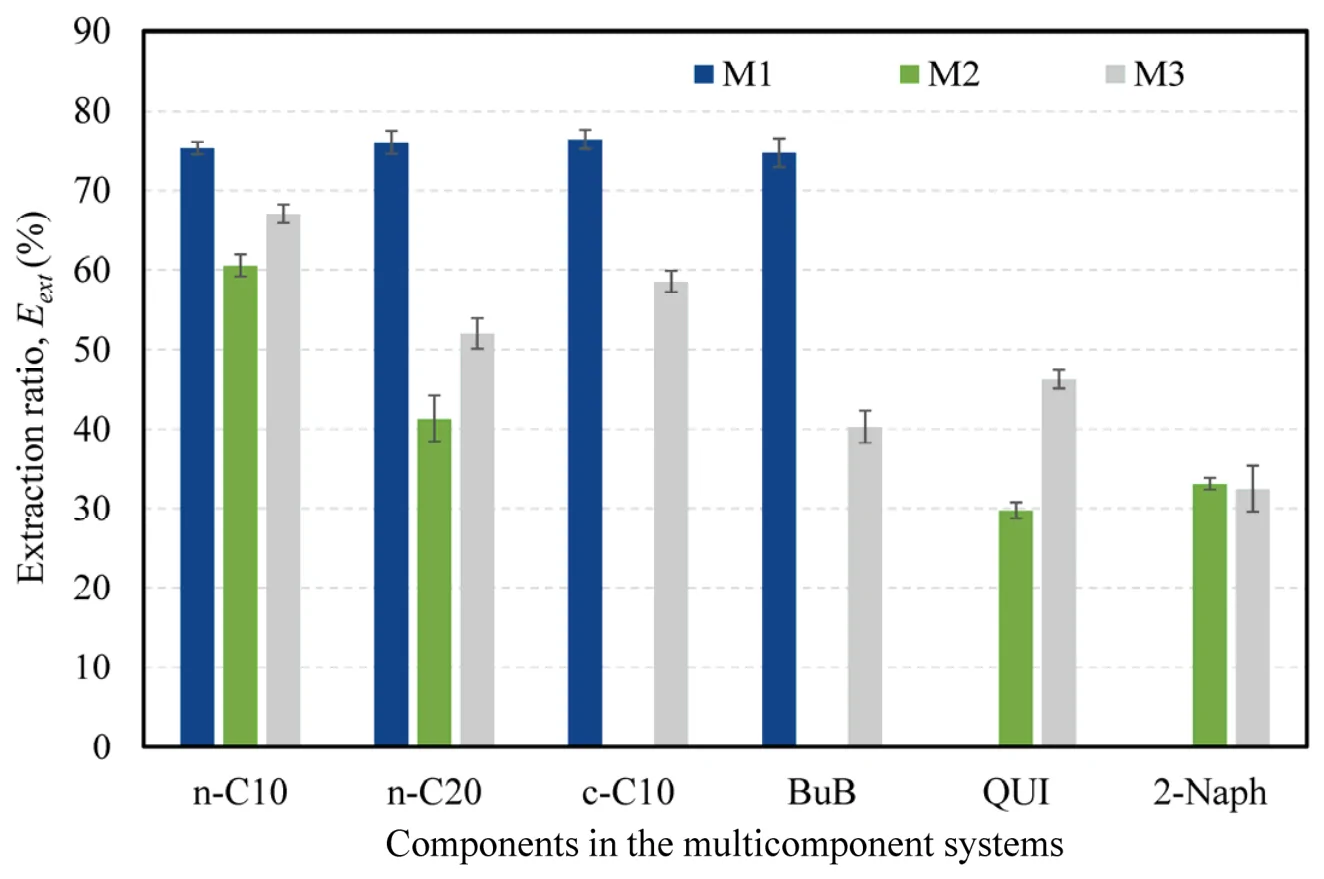
Component extraction ratios in the multicomponent oil systems. M1 contained n-C10, n-C20, c-C10, and BuB; M2 contained n-C10, n-C20, QUI, and 2-Naph; and M3 contained n-C10, n-C20, c-C10, BuB, QUI, and 2-Naph. The error bars represent the standard deviations from three independent simulations.

The multicomponent systems displayed distinct component extraction patterns, ranging from a relatively uniform response in M1 to more differentiated behavior in M2 and M3. As shown in Figure 7 and Table S5, the extraction ratios of the four components in M1 were closely distributed between 74.71% and 76.40%, indicating limited differentiation among these components under the investigated composition. In M2, the extraction ratios ranged from 29.75% to 60.53%, following the order n-C10 > n-C20 > 2-Naph > QUI. M3 exhibited a similarly broad range of 32.46–67.05%, with the extraction ratios decreasing in the order n-C10 > c-C10 > n-C20 > QUI > BuB > 2-Naph. Thus, M1 showed comparatively balanced extraction, whereas M2 and M3 exhibited more pronounced component selectivity. Because both the component identities and their fractions differed among the three systems, these contrasts are more appropriately interpreted as responses to the overall composition rather than as an isolated effect of the number of components.

Comparisons of common components across the multicomponent systems further indicate that their extraction was influenced by the surrounding composition. The average extraction ratios of n-C10 were 75.28%, 67.05%, and 60.53% in M1, M3, and M2, respectively, while the corresponding values for n-C20 were 76.04%, 51.99%, and 41.28% (Figure 7 and Table S5). Other shared components exhibited different responses. The extraction ratio of BuB decreased from 74.71% in M1 to 40.27% in M3, whereas that of QUI increased from 29.75% in M2 to 46.20% in M3. By comparison, the values of 2-Naph in M2 and M3 were relatively close, at 33.08% and 32.46%, respectively. These contrasting responses suggest that changes in the overall composition were not associated with a uniform increase or decrease in extraction across all components. The standard deviations from the three independent simulations ranged from 0.29% to 1.16% and were smaller than the major differences observed between systems, supporting the reproducibility of the principal extraction trends. The resulting component rankings are therefore consistent with composition specific competition among coexisting oil components.

Taken together, the binary and multicomponent results indicate that extraction from mixed oil systems reflects the combined influences of composition, molecular mobility, and component type. The binary systems showed a notable contrast between nonpolar and polar components. Higher proportions of the nonpolar components C10iso and BuB were accompanied by increased total extraction ratios, whereas increasing the proportions of the polar components DecA, QUI, and 2-Naph resulted in lower total extraction ratios, with the decreases being particularly evident in the QUI and 2-Naph systems. Although this contrast does not establish polarity as the sole controlling property, it suggests that molecular polarity and functional groups can alter the transfer environment within mixed oil systems. The temporal profiles of extracted molecule numbers and the relative diffusion coefficients further showed that these extraction differences were accompanied by variations in molecular mobility, although mobility alone did not provide a quantitative description of the observed selectivity. The multicomponent results also demonstrated that the extraction characteristics of a given component varied with the identities and proportions of the surrounding components. These observations are consistent with a composition-dependent balance between cohesive interactions among oil molecules and interactions between CO_2_ and the oil components. In particular, polar functional groups may promote specific associations within the oil phase, whereas the behavior of nonpolar components is more closely related to dispersion interactions and their accessibility to CO_2_. The energetic and structural basis of these component type effects is examined further in Section 2.4.

### 2.4. Energetic and Structural Basis of Component-Specific CO_2_ Extraction: Competition Between Oil–Oil Cohesion and CO_2_–Oil Interactions

The energetic competition between oil–oil cohesion and CO_2_–oil interaction was characterized using the gas–oil interaction competition factor, *R_comp_*:

(10) $$R_{comp}=\frac{\left|{\bar{E}}_{CO_{2}–oil}\right|/N_{oil}}{\left|{\bar{E}}_{oil–oil}\right|/N_{oil}}$$

**Figure 8:**
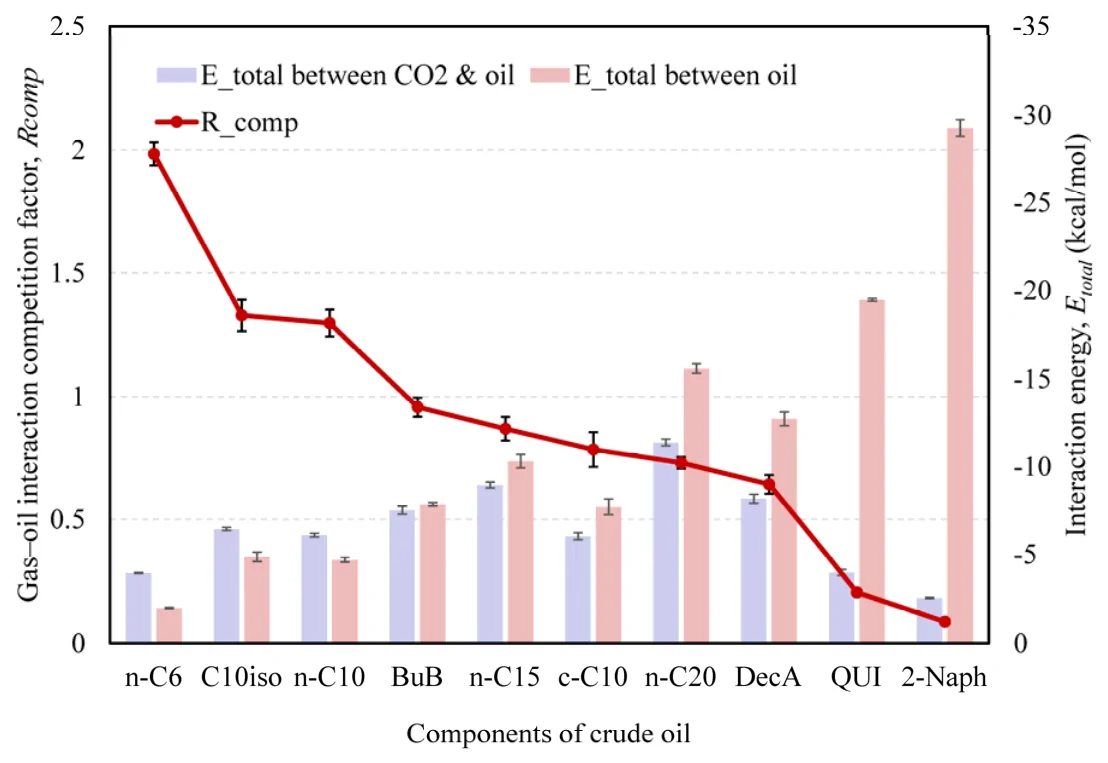
Energetic competition between oil–oil cohesion and CO_2_–oil interaction in the single-component systems.

Under this definition, a larger *R_comp_* indicates a greater relative contribution of CO_2_–oil attraction, whereas a smaller value indicates a stronger energetic bias toward oil–oil cohesion. Across the investigated components as shown in Figure 8, *R_comp_* decreased from 1.985 for n-C6 to 0.087 for 2-Naph, and its descending order was identical to that of the single-component extraction ratios. The three components with *R_comp_* > 1 (n-C6, C10iso, and n-C10) exhibited extraction ratios of 80.70%, 64.82%, and 64.43%, respectively. BuB had nearly balanced CO_2_–oil and oil–oil interaction magnitudes, with *R_comp_* = 0.957 and an extraction ratio of 52.73%. This correspondence is interpreted as an energetic description of the observed extraction trend within the present component set, rather than as evidence that *R_comp_* is an independent predictor or that *R_comp_* = 1 represents a universal extraction threshold. 

The energetic correspondence was essentially unchanged after excluding 2-Naph. The Pearson coefficient between *R_comp_* and extraction ratio changed only from 0.951 to 0.948, while the Spearman coefficient remained 1.000 (Table S2). The observed energetic ordering was therefore not controlled by the 2-Naph endpoint.

The n-alkane series further illustrates how the relative growth of the two interaction terms corresponds to the extraction tendency. From n-C6 to n-C20, *R_comp_* decreased successively from 1.985 to 0.730, accompanied by a decrease in extraction ratio from 80.70% to 48.63%. The absolute value of interaction energies of CO_2_–oil increased from 3.96 kcal/mol to 11.39 kcal/mol over the same series, indicating that the reduced extraction of longer n-alkanes did not arise from a weakening of the absolute CO_2_–oil attraction. Instead, the oil–oil interaction energies increased more rapidly, from 1.99 kcal/mol for n-C6 to 15.60 kcal/mol for n-C20, and the energetic balance shifted toward oil-phase cohesion. Longer hydrocarbon chains generally provide more cumulative dispersion contacts and stronger cohesive interactions [21]. Molecular geometry produced a related effect at constant carbon number: n-C10 and c-C10 had nearly identical CO_2_–oil interaction energies of 6.10 kcal/mol and 6.04 kcal/mol, respectively, whereas the magnitude of the c-C10 oil–oil interaction was approximately 1.64 times that of n-C10. Correspondingly, *R_comp_* decreased from 1.298 to 0.784 and the extraction ratio decreased from 64.82% to 49.25%. Therefore, the lower extraction of the longer-chain and cyclic hydrocarbons was more consistent with the increasing relative contribution of oil–oil cohesion than with diminished CO_2_–hydrocarbon contact.

A stronger energetic bias toward oil–oil cohesion was observed for DecA, QUI, and 2-Naph. Their *R_comp_* values were 0.642, 0.205, and 0.087, respectively, corresponding to extraction ratios of 26.87%, 24.16%, and 5.85%. For QUI, the magnitude of the oil–oil interaction was approximately 4.88 times that of the CO_2_–oil interaction, whereas the corresponding ratio reached approximately 11.47 for 2-Naph. However, the numerical differences in *R_comp_* were not proportional to the differences in extraction ratio. For example, *R_comp_* decreased substantially from DecA to QUI, whereas their extraction ratios differed by only 2.71%. The low extraction ratios, together with the representative associated configurations discussed below and molecular configurations in Figure 1, suggest that a large fraction of these molecules remain within compact oil-associated structures, thereby limiting molecular or functional-site exposure to CO_2_. The CO_2_–oil energy consequently contains contributions from both the strength of individual contacts and the extent of actual molecular accessibility. Therefore, molecular accessibility may contribute to the non-proportional numerical relationship between *R_comp_* and extraction ratio. The observed ordering of *R_comp_* should therefore be regarded as a qualitative energetic correspondence within the present systems rather than a quantitative or independently validated relationship with the extraction ratios.

**Figure 9:**
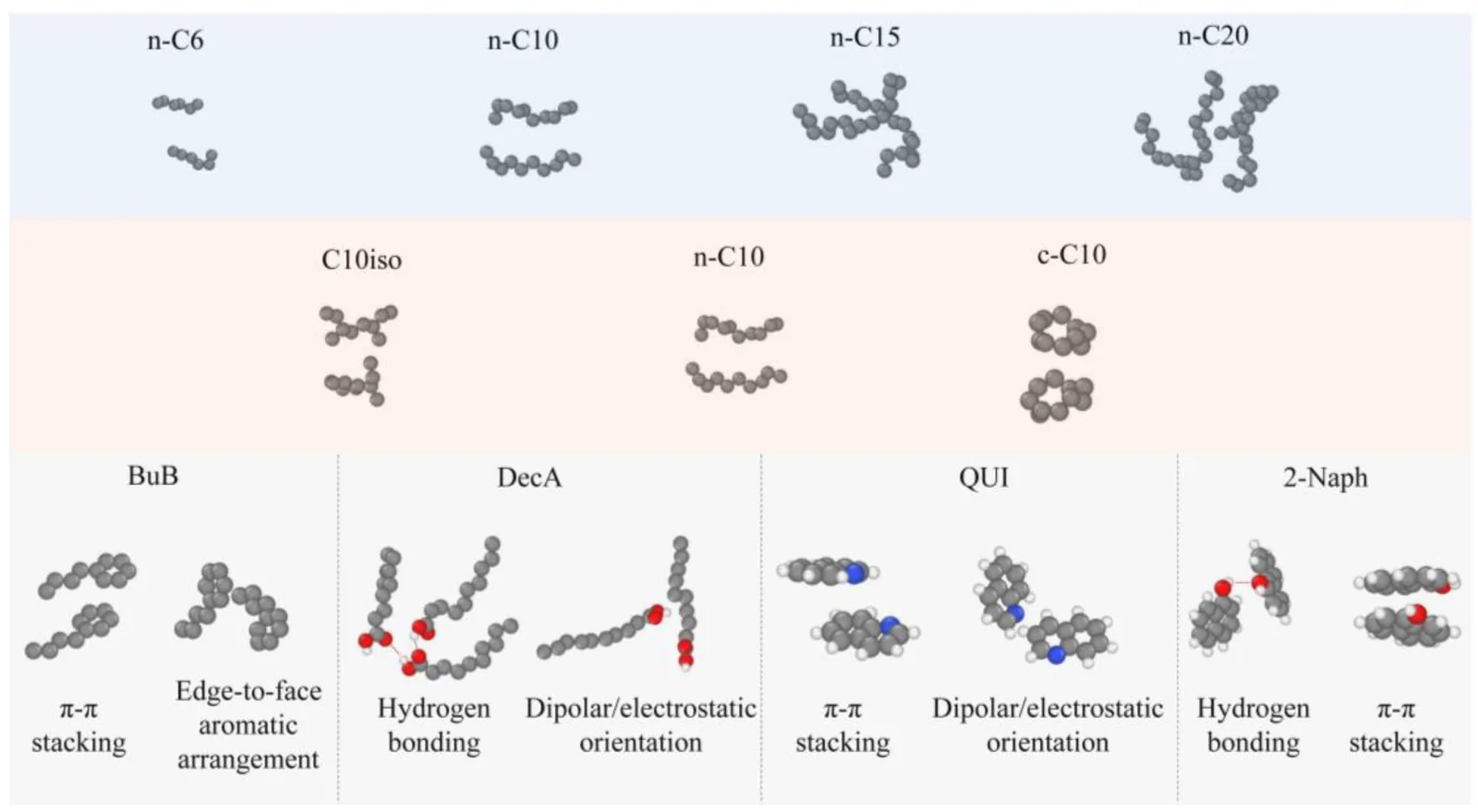
Representative characteristic association modes in the pure crude-oil component systems. Additionally, dispersion interactions are present in all systems.

The representative molecular configurations from the simulations in Figure 9 illustrate that the origin of oil–oil cohesion differs among molecular classes. The associations of n-C6, n-C10, n-C15, n-C20, C10iso, and c-C10 were dominated by dispersion interactions. Increasing chain length provided more extensive intermolecular contact, whereas the cyclic geometry of c-C10 may permit relatively compact molecular packing. The aromatic and polar components displayed representative arrangements consistent with additional directional association modes. Aromatic π–π stacking, edge-to-face arrangements, and dipolar/electrostatic orientations can provide additional directional contributions to molecular association. Specifically, BuB showed configurations consistent with π–π stacking and edge-to-face aromatic arrangements; DecA displayed O–H···O hydrogen-bonded and dipolar/electrostatic orientations; QUI exhibited arrangements consistent with π–π stacking and dipolar/electrostatic orientation; and 2-Naph combined representative O–H···O hydrogen bonding with π–π stacking. These configurations are consistent with the comparatively strong oil–oil interaction energies of DecA, QUI, and especially 2-Naph, although individual snapshots cannot quantify the energetic contribution of each association mode. The accumulation of dispersion contacts and additional directional associations may therefore stabilize oil-associated structures and reduce the tendency of molecules to transfer into the CO_2_-rich region.

**Figure 10:**
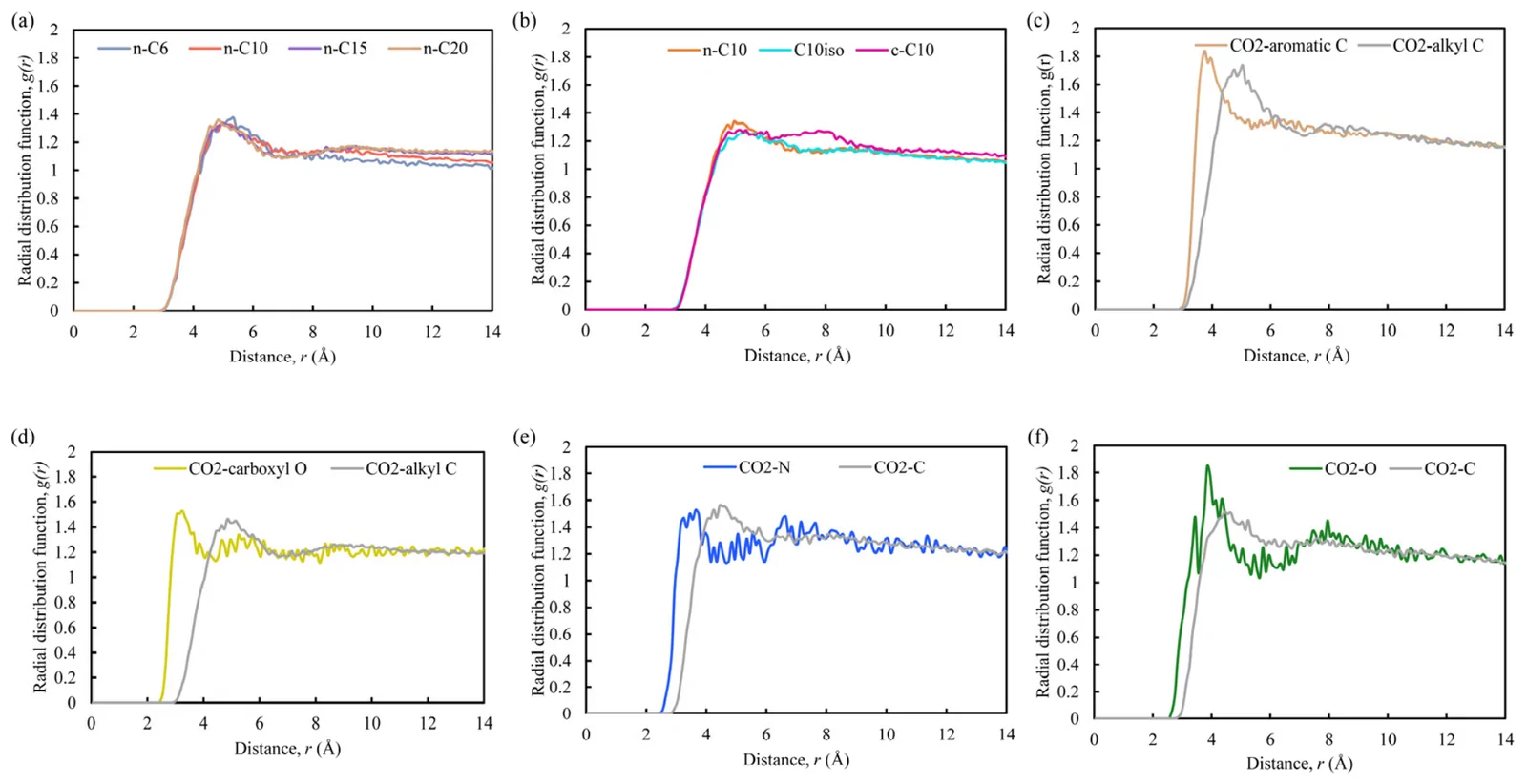
Radial distribution functions between the C atom of CO_2_ and selected atoms of the oil molecules in the uniformly dispersed CO_2_-rich-phase models: (**a**) C of n-C6, n-C10, n-C15, and n-C20; (**b**) C of n-C10, C10iso, and c-C10; (**c**) aromatic and alkyl C of BuB; (**d**) carbonyl O and alkyl C of DecA; (**e**) N and aromatic C of QUI; and (**f**) hydroxyl O and aromatic C of 2-Naph. (The RDF peaks indicate characteristic short-range spatial correlations between the selected sites, but are not used alone to quantify interaction strength or thermodynamic preference).

The local spatial distribution of CO_2_ around the hydrocarbons is characterized in Figure 10, using models in which the influence of macroscopic oil aggregation was minimized by dispersing the oil molecules in the CO_2_-rich phase. For n-C6, n-C10, n-C15, and n-C20, the principal CO_2_-carbon peaks were located at 5.0 Å, with comparable maximum values of 1.3–1.4. The similar peak positions and heights indicate that the primary local CO_2_ distribution around alkane carbon sites was only weakly affected by chain length. The main peaks of n-C10, C10iso, and c-C10 also occurred within a similar range of 5.0 Å and exhibited comparable heights. The broad feature of c-C10 at approximately 7–8 Å is attributable to the geometric distribution of carbon sites within the cyclic molecule, rather than a distinct short-range interaction. Because the principal peak position and height of c-C10 remained close to those of n-C10 and C10iso, no clear change was observed in its primary local CO_2_ distribution. Hence, the extraction difference between n-C10 and c-C10 is more consistently explained by the stronger oil–oil cohesion of c-C10 than by a change in its local CO_2_ distribution.

Distinct site selectivity was observed for the aromatic and polar components in Figure 10c–f. In BuB, the first C_CO2_-C_aromatic_ peak occurred at 3.75 Å, whereas the corresponding C_CO2_-C_alkyl_ peak appeared at 5.05 Å, indicating preferential short-range CO_2_ distribution near the aromatic region. For DecA, the C_CO2_-C_carbonyl_ distribution developed a peak at approximately 3.25 Å, preceding the main C_CO2_-C_alkyl_ peak at 4.85 Å. In QUI, the first C_CO2_-N feature appeared at 3.65 Å, while the main distribution around aromatic carbon sites occurred at 4.45 Å. Similarly, 2-Naph exhibited a distinct short-range C_CO2_-O_hydroxyl_ peak at 3.85 Å, compared with an aromatic-carbon peak at approximately 4.65 Å. These RDF results characterize preferential CO_2_ enrichment around specific molecular sites. In particular, the local enrichment around the carbonyl O of DecA, the N site of QUI, and the hydroxyl O of 2-Naph coexisted with their low overall extraction ratios. Local site enrichment alone was therefore insufficient to account for overall extraction, and strong oil–oil association may reduce the accessibility of these favorable CO_2_-contact sites within the condensed oil structure. 

**Figure 11:**
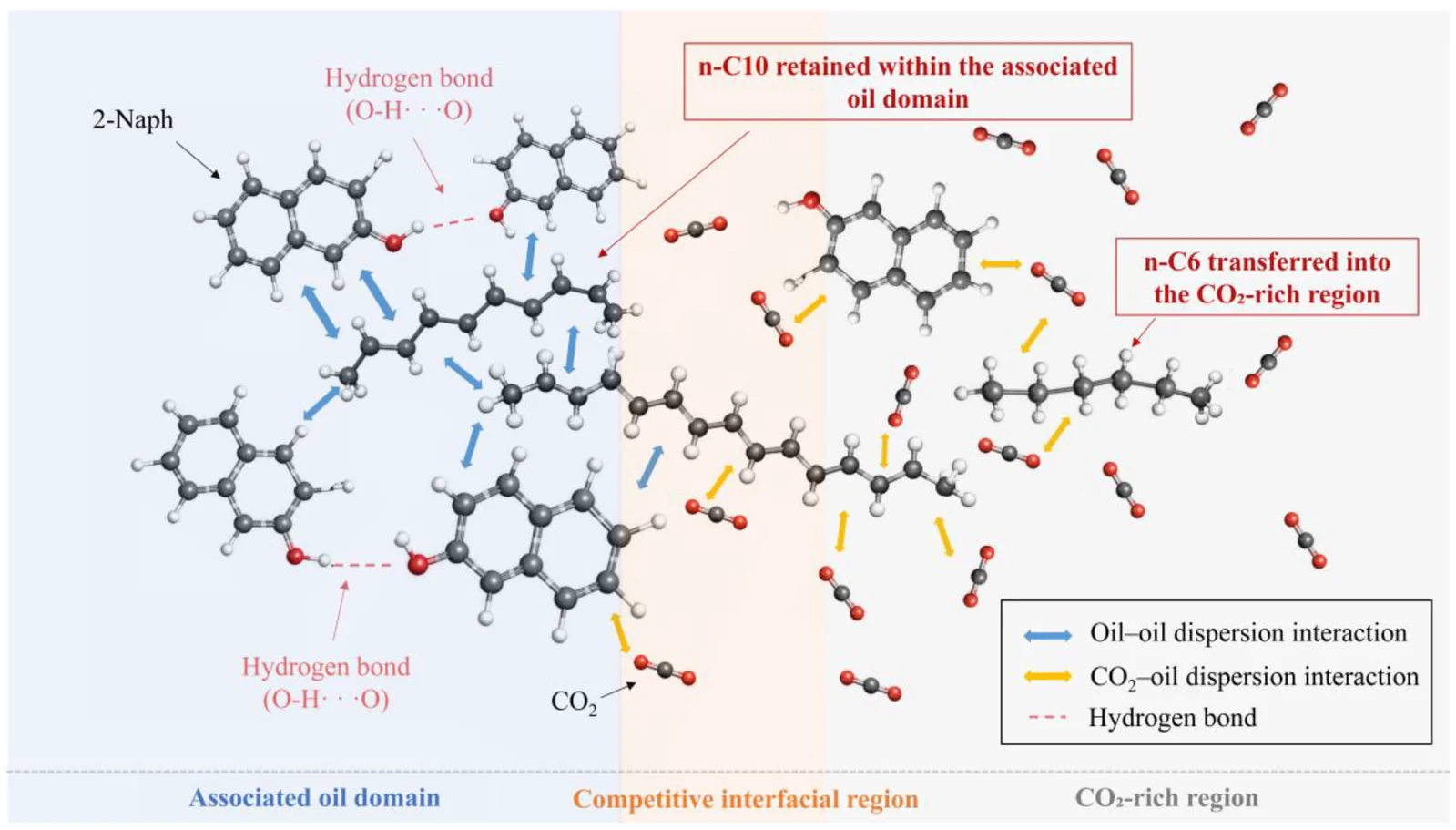
Conceptual schematic of the competition between oil–oil cohesion and CO_2_–oil association during the transfer of crude-oil components from the associated oil domain to the CO_2_-rich region.

As a conceptual synthesis of the preceding results, Figure 11 summarizes the competition between oil–oil cohesion and CO_2_–oil interaction across three characteristic regions. Within the associated oil domain, hydrogen-bonded and dispersion-stabilized molecular arrangements retain hydrocarbons through multiple oil–oil contacts. In the competitive interfacial region, oil–oil and CO_2_–oil interactions coexist, and the transfer tendency is associated with their relative energetic contributions and with molecular accessibility to CO_2_. When CO_2_–oil association becomes relatively favorable and an oil molecule is sufficiently exposed, transfer into the CO_2_-rich region may become more likely. This conceptual framework integrates the energetic and structural characteristics in Section 2.4 to provide a molecular basis for interpreting the composition-dependent and component selective transfer characteristics identified in Section 2.3. Overall, the observed component-specific transfer characteristics are consistent with a balance between local CO_2_–oil association and collective oil–oil cohesion, while molecular association may regulate the accessibility of favorable CO_2_-contact sites.

## 3. Model and Methodology

### 3.1. Model Construction and Simulation Details

**Figure 12:**
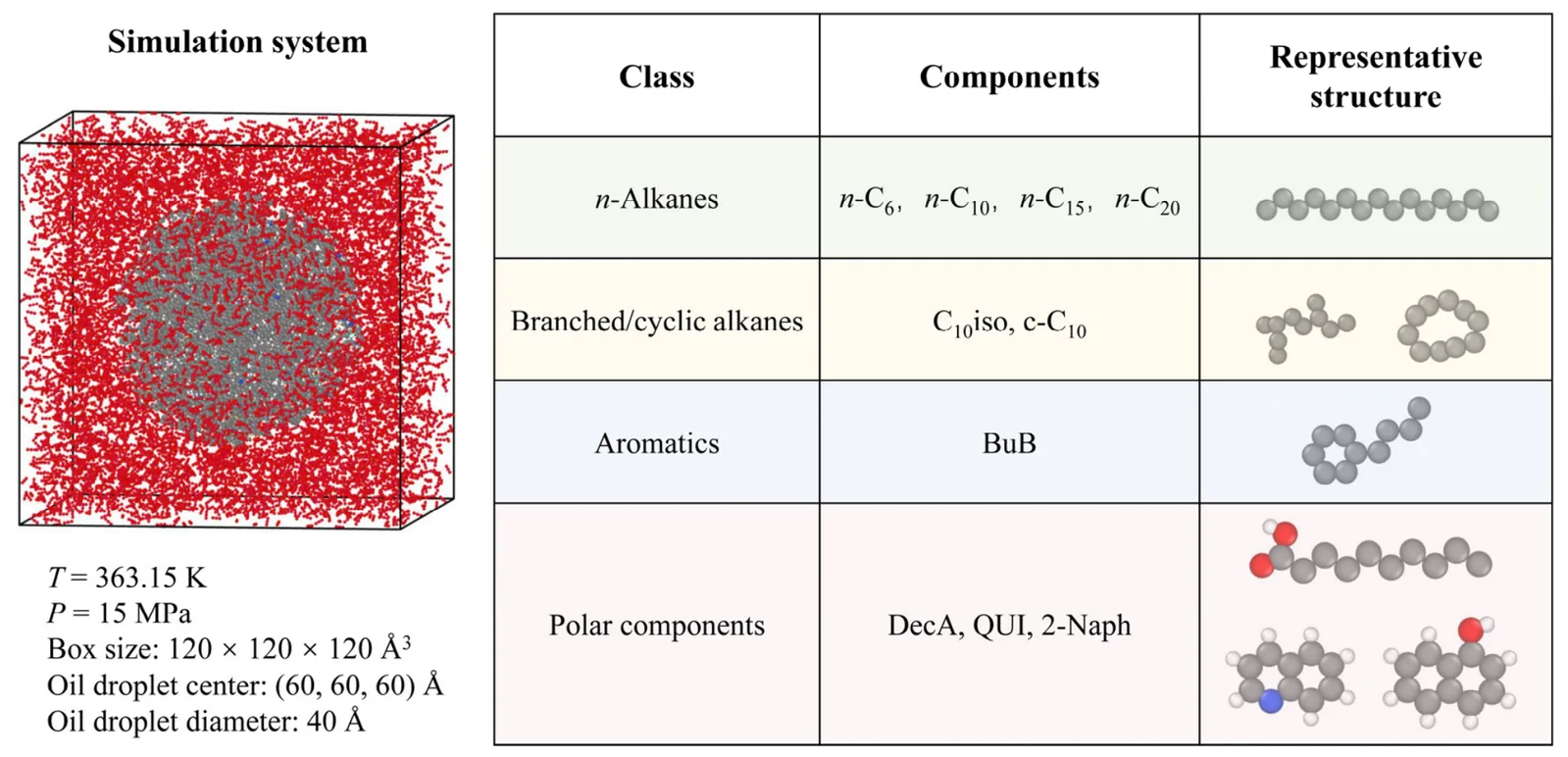
Simulation model and molecular structures for evaluating the extraction of crude-oil components by supercritical CO_2_.

Molecular dynamics simulations were performed using the Large-scale Atomic/Molecular Massively Parallel Simulator (LAMMPS, version 29 August 2024) to investigate the extraction behavior of supercritical CO_2_ toward components of crude oil. As illustrated in Figure 12, the simulation system consisted of a spherical oil droplet surrounded by a continuous CO_2_ phase in a cubic simulation box with an edge length of 120 Å. The initial oil droplet was centered at (60, 60, 60) Å and had a radius of 40 Å. The remaining volume was occupied by CO_2_ molecules, and periodic boundary conditions were applied in all three spatial directions. The initial system configurations were generated using PACKMOL (version 18.169) with a minimum intermolecular tolerance of 3.0 Å to avoid severe atomic overlap.

Ten representative model-oil components were considered, including n-hexane (n-C6), n-decane (n-C10), n-pentadecane (n-C15), n-eicosane (n-C20), 3,6-dimethyloctane (C10iso), cyclodecane (c-C10), butylbenzene (BuB), quinoline (QUI), decanoic acid (DecA), and 2-naphthol (2-Naph). These molecules were selected to represent differences in carbon number, molecular size, chain branching, cyclic structure, aromaticity, heteroatom functionality, and polarity. The molecular compositions and molecule numbers, determined from the respective densities of the single-component systems are summarized in Table 3. Each simulation system contained 7424 CO_2_ molecules from the actual gas equation of state at 363.15 K and 15 MPa. It should be noted that the binary and multicomponent systems were constructed with a fixed total of 784 oil molecules rather than at equal oil density or equal oil volume. Their extraction ratios were therefore used to examine changes with composition within the mixed oil models and were not directly compared with those of the systems containing a single component. These ratios describe molecular transfer over the simulated time scale and should not be interpreted as density normalized equilibrium partitioning data.

In addition, ten auxiliary simulations were constructed for RDF analysis, corresponding to the ten single-component systems. Unlike the droplet models, the oil and CO_2_ molecules were initially distributed uniformly throughout the simulation box. The molecular composition, number of molecules, simulation-box dimensions, force-field parameters, temperature, and simulation protocol were otherwise identical to those of the corresponding single-component systems. These auxiliary models were used exclusively for RDF analysis and were not used to calculate the extraction ratio.

To examine component-coupling effects in multicomponent oils, binary oil droplets containing n-C10 and a second model-oil component were constructed. The second components included nonpolar components (C10iso and BuB) and polar components (DecA, QUI, and 2-Naph). For each binary combination summarized in Table 3, n-C10-to-partner molar ratios of 8:2, 7:3, and 5:5 were considered. The total number of oil molecules was maintained at 784.

All fluid molecules were described using the TraPPE (Transferable Potentials for Phase Equilibria) force-field family. A united-atom representation was adopted for CO_2_-free hydrocarbon groups in n-C6, n-C10, n-C15, n-C20, C10iso, c-C10, BuB, and DecA [27,28,29], whereas all-atom representations were used for QUI and 2-Naph [30]. CO_2_ was represented by a rigid three-site TraPPE model. The molecular structures of all investigated molecules are shown in Figure 12. The complete nonbonded parameters and partial charges, bond-stretching parameters, angle-bending parameters, and dihedral parameters used in the simulations are provided in Tables S6–S9 of the Supplementary Information, respectively.

The nonbonded interaction energy between interaction atoms *a* and *b* was expressed as the sum of the Lennard–Jones 12–6 and Coulombic contributions:

(11) $$E=E_{LJ}+E_{coul}=4\varepsilon_{ab}\left[{\left(\frac{\sigma_{ab}}{r_{ab}}\right)}^{12}-{\left(\frac{\sigma_{ab}}{r_{ab}}\right)}^{6}\right]+\frac{q_{a}q_{b}}{4\pi \varepsilon_{0}r_{ab}}$$

where *r_ab_* is the distance between interaction atoms *a* and *b. ε_ab_* and *σ_ab_* are the corresponding Lennard–Jones well depth and zero-potential distance, respectively. *q_a_* and *q_b_* are the partial charges of atom *a* and *b*, respectively. *ε*_0_ is the dielectric vacuum permittivity.

A cutoff distance of 14 Å was applied to the short-range Lennard–Jones and real-space electrostatic interactions. Long-range electrostatic interactions were evaluated using the particle–particle particle–mesh method with a relative accuracy of 10^−4^. For unlike Lennard–Jones site pairs, the cross parameters were determined consistently using arithmetic mixing for the size parameter and geometric mixing for the energy parameter. Intramolecular Lennard–Jones and Coulombic interactions for 1–2, 1–3, and 1–4 bonded pairs were excluded using the command of special_bonds lj/coul 0.0 0.0 0.0. The energy minimization was performed using the conjugate-gradient algorithm with energy and force tolerances of 1.0 × 10^−5^ and 1.0 × 10^−7^, respectively, a maximum of 10,000 minimization iterations, and a maximum of 100,000 force/energy evaluations. The temperature was controlled using a Nosé–Hoover thermostat with a temperature damping parameter of 500 fs. The production simulations were performed in the NVT ensemble with a time step of 1 fs for 1 ns, divided into system relaxation and equilibration period. The temporal evolutions of the molecule numbers of extracted oil and dissolved CO_2_, interaction energy and system pressure were monitored to evaluate the dynamic stability of the systems, as showed in Figure S3. Due to the fluctuation of these quantities without a persistent temporal drift, the range of 800–1000 ps were treated as equilibration period.

### 3.2. Calculation and Analysis Methods

For systems containing a single oil component, the extraction calculation retained a spherical boundary with a radius of 40 Å. The instantaneous center of mass of all oil molecules was used as the center of the counting sphere, and additional calculations with radii of 37 and 43 Å were performed only to evaluate the boundary sensitivity of these systems (Figure S1). For each independent trajectory of the binary and multicomponent systems, the extraction boundary was determined from the radial mass density profile of the total oil during 800–1000 ps. The radial profile was calculated relative to the instantaneous mass weighted center of all oil molecules. The inner reference density was averaged within 2 Å on either side of the radius corresponding to the maximum density, whereas the outer reference density was averaged over the radial range of 56–60 Å. The extraction boundary was defined as the first outward position beyond the density maximum at which the total oil mass density reached the midpoint between the two reference densities. An oil molecule was counted as extracted when its molecular center of mass was located outside the boundary determined for the corresponding trajectory. The resulting boundary radii are reported in Table S4. The extraction ratio of component *i*, denoted as *E_ext_*, was calculated as

(12) $$E_{ext}=\frac{N_{i,out}(t)}{N_{i,0}}\times 100\%$$

where *N_i_*_,*out*_(*t*) is the number of molecules of component *i* outside the boundary determined for the corresponding system and *N_i_*_,0_ is its initial molecular number. As shown in Figure S3, the extracted-molecule number, dissolved CO_2_ population, interaction energy, and system pressure exhibited substantially reduced temporal drift during the late stage of the simulations. The 800–1000 ps interval was therefore selected as a statistically stationary sampling window.

Molecular mobility was evaluated using the mean squared displacement (*MSD*) and self-diffusion coefficient (*D*), obtained from Equation (13) [18]:

(13) $$D=\frac{1}{6}\frac{dMSD(t)}{dt}=\frac{1}{6}\frac{d\langle \left|r_{i}(t_{0}+t)-r_{i}{\left(t_{0}\right)}^{2}\right|\rangle }{dt}$$

where *r_i_*(*t*) is the position of molecule *i* at time *t*, and the angular brackets denote an ensemble average. The diffusion analysis was performed for oil molecules located outside a sphere with a fixed radius of 40 Å. Although the extraction ratios of the binary systems were recalculated using boundaries obtained from their density profiles, most of these boundaries were smaller than 40 Å. The fixed radius therefore selected molecules located farther from the oil rich domain in most systems and was retained to characterize molecular mobility in the outer region of the CO_2_ phase. The resulting diffusion coefficients and their ratios were used as qualitative indicators of relative molecular mobility rather than as quantitative predictors of extraction. Molecular trajectories were processed using unwrapped coordinates, and the instantaneous center of mass of all oil molecules was used as the reference center to reduce the contribution of collective droplet translation. The diffusion coefficients were obtained by linear fitting of the approximately diffusive portions of the MSD curves within 800–1000 ps. Representative MSD curves for the binary systems are provided in Figure S4.

The CO_2_–oil interaction energy, *E_CO_*_2–*oil*_, was calculated as the intermolecular nonbonded interaction energy between the complete CO_2_ population and the complete oil population in each single-component system, whereas *E_oil–oil_* represents the intermolecular nonbonded interaction energy among the oil molecules. Both terms include the Lennard–Jones and Coulombic contributions defined in Equation (11). Because the numbers of oil molecules differed among the single-component systems, the time-averaged interaction energies were normalized by the corresponding number of oil molecules, *N_oil_*, and are reported on a per-oil-molecule basis. The interaction energies were averaged over the statistically stationary interval of 800–1000 ps.

Radial distribution function (*g*(*r*)), used to characterize the local molecular association, represents the relative probability of finding interaction atom *b* at a distance *r* from reference atom *a*. The peak position reflects the distance, whereas the peak height indicates the degree of local accumulation. Atomic mass centers were selected according to the specific intermolecular interaction under investigation. 

### 3.3. Model Validation and Statistical Analysis

Density and solubility parameters reflect intermolecular interactions and can be used to evaluate the reliability of the simulation system. The simulated equilibrium densities of CO_2_ and the oil components were compared with experimental reference data from National Institute of Standards and Technology (NIST) and Hansen Solubility Parameters in Practice (HSPIP, version 6.1.02), respectively in Table 4 [17]. In addition, the solubility parameters of CO_2_ and the oil components obtained from molecular simulations were additionally compared in Section 2.2.

Three independently initialized simulations were performed for the single-component, binary, and multicomponent extraction systems. Unless otherwise specified, each reported extraction characteristic represents the mean of three independent trajectories, with the final 800–1000 ps interval of each trajectory used for temporal averaging. The individual extraction ratios from the three independent simulations, together with their averages and standard deviations, are summarized in Tables S1, S3 and S5 of the Supplementary Information. For the binary and multicomponent systems, the boundary radii determined independently from the three trajectories, together with their averages and standard deviations, are reported in Table S4. Standard deviations (SD) among the three independent trajectory averages were used to characterize run-to-run uncertainty. Independent replicate simulations were also performed for the MD-derived quantities in Figure 3a and the diffusion-coefficient ratios in Figure 6. For the interaction-energy quantities in Figure 8, statistical uncertainty was evaluated using block averaging over the stationary sampling interval.

The present simulations are intended for comparative evaluation of component-dependent molecular transfer under a common system geometry. A systematic convergence analysis with respect to droplet and simulation-box dimensions was not performed; therefore, the absolute extraction values should not be interpreted as size-independent macroscopic extraction ratios.

The pure-component property validation was used to verify the implementation and basic thermophysical consistency of the adopted molecular models. No mixture-specific cross-interaction parameters were refitted in this work; instead, the literature-based TraPPE parameters and the same combining rule were consistently applied to all systems. Accordingly, the results for the polar components are primarily interpreted in terms of comparative molecular trends under the common simulation framework rather than as quantitative predictions of mixture phase-equilibrium properties.

## 4. Conclusions

The single-component simulations showed that molecular architecture and chemical functionality differentiated extraction behavior more effectively than molecular size alone under the investigated conditions. At 363.15 K and 15 MPa, the extraction ratios ranged from 80.70% for n-C6 to 5.85% for 2-Naph. Although extraction decreased from n-C6 to n-C20 within the n-alkane series, the six components containing ten carbon atoms exhibited a much broader range of 5.85–64.82%. The differences among linear-, branched-, cyclic-, aromatic-, and heteroatom-containing molecules indicate that topology, aromaticity, and polar functional groups need to be considered together with carbon number and molar mass when interpreting component extraction.

The solubility parameter analysis provided a thermodynamic basis for comparing the single-component extraction characteristics. The MD results for supercritical CO_2_ agreed with estimates derived from NIST data, and the empirical correlation *δ_CO_*_2_ = 7.28*ρ_r_* captured most of the combined temperature and pressure dependence over 303.15–363.15 K and the investigated reduced density range. A residual temperature dependence remained and was not represented explicitly in this simplified correlation. The extraction ratio generally decreased as the oil–CO_2_ solubility parameter difference increased, and this negative correspondence persisted after 2-Naph was excluded. The solubility parameter difference can therefore serve as a comparative thermodynamic descriptor within the investigated component set.

The mixed oil simulations showed that component transfer depended on both the identity and proportion of the surrounding components. In the binary systems evaluated using oil boundaries determined by the half density criterion, increasing the proportions of the nonpolar partners C10iso and BuB was associated with higher total extraction ratios, whereas increasing the proportions of DecA, QUI, and 2-Naph was associated with lower values. The multicomponent systems likewise showed redistribution that depended on the overall composition rather than a uniform increase or decrease across all components. M1 exhibited a relatively narrow extraction range of 74.71–76.40%, whereas M2 and M3 showed more differentiated component responses. For example, the extraction ratio of BuB decreased from 74.71% in M1 to 40.27% in M3, while that of QUI increased from 29.75% in M2 to 46.20% in M3. Relative diffusion coefficients qualitatively reflected differences in molecular mobility but did not independently predict extraction. Because the mixed systems were constructed using a fixed total number of oil molecules, these results describe composition-dependent transfer within the present model framework and are not interpreted as density-normalized equilibrium partitioning data.

The energetic and structural analyses connected the observed extraction characteristics to competition between CO_2_–oil association and oil–oil cohesion. The gas–oil interaction competition factor, *R_comp_*, decreased from 1.985 for n-C6 to 0.087 for 2-Naph and followed the extraction ratio ranking within the investigated component set. This correspondence remained after excluding 2-Naph, indicating that the energetic ordering was not controlled by this endpoint. The factor is therefore interpreted as a comparative energetic descriptor rather than an independent quantitative predictor or a universal extraction threshold. Molecular configurations and RDFs showed that association among nonpolar hydrocarbons was dominated by dispersion interactions, whereas hydrogen bonding, aromatic stacking, and dipolar or electrostatic organization provided additional cohesive contributions in polar and aromatic components. The simulated 2-Naph rich domain displayed noncrystalline, disordered organization with liquid-like short-range correlations, although the RDFs did not distinguish among an amorphous state, a metastable liquid, or a state plasticized by CO_2_. Moreover, preferential CO_2_ enrichment near the aromatic or polar sites of BuB, DecA, QUI, and 2-Naph did not by itself account for their overall extraction characteristics. Collectively, the results support a molecular framework in which selective component transfer reflects a balance between local CO_2_–oil association and collective oil–oil cohesion, with molecular mobility, mixture composition, and the accessibility of favorable CO_2_ contact sites jointly modifying this balance.

## Figures and Tables

**Table 1:** Comparison of MD-derived and Hansen solubility parameters of crude oil components at 298.15 K and 1 MPa.

	*CED_MD_* (MPa)	*δ_MD_* (MPa^1/2^)	*δ_Hansen_* (MPa^1/2^)	Relative Deviation (%)
n-C6	180.32	13.43	14.9	9.87
C10iso	207.45	14.4	15.2	5.26
n-C10	213.55	14.61	15.7	6.94
BuB	309.27	17.59	17.44	−0.86
n-C15	231.53	15.22	15.7	3.06
c-C10	247.12	15.72	17.17	8.44
n-C20	240.89	15.52	16.5	5.94
DecA	341.98	18.49	18.68	1.02
QUI	482.42	21.96	22	0.18
2-Naph	540.25	23.24	23.24	0.00

**Table 2:** Linear correlation between the CO_2_ solubility parameter and reduced density at different temperatures.

Temperature (K)	Number of Data Points (n)	*ρ_r_* Range	*k_T_* (MPa^0.5^)	*R_T_* ^2^	MAPE of Individual Fit (%)	MAPE Using *k_CO_*_2_ = 7.28 (%)
363.15	10	0.427–1.963	7.064	0.999	1.49	4.01
333.15	9	0.618–2.124	7.299	0.997	1.42	1.40
303.15	9	1.645–2.279	7.384	0.999	0.25	1.46
All data	28	0.427–2.279	7.280	0.995	—	2.35

Note: *k_T_* denotes the temperature-specific slope obtained by least-squares regression constrained through the origin, whereas *k_CO_*_2_ is the unified slope obtained from all 28 data points. MAPE represents the mean absolute percentage error.

**Table 3:** Molecular compositions of the simulation systems.

System Type	System ID	Oil Component	Molar Ratio	Number of Oil Molecules
Single-component	S1	n-C6	/	1154
	S2	n-C10	/	784
	S3	n-C15	/	557
	S4	n-C20	/	431
	S5	C10iso	/	865
	S6	c-C10	/	937
	S7	BuB	/	1011
	S8	QUI	/	1319
	S9	DecA	/	803
	S10	2-Naph	/	1236
Binary	B1	n-C10/C10iso	8:2	627:157
	B2	n-C10/C10iso	7:3	548:236
	B3	n-C10/C10iso	5:5	392:392
	B4	n-C10/BuB	8:2	627:157
	B5	n-C10/BuB	7:3	548:236
	B6	n-C10/BuB	5:5	392:392
	B7	n-C10/DecA	8:2	627:157
	B8	n-C10/DecA	7:3	548:236
	B9	n-C10/DecA	5:5	392:392
	B10	n-C10/QUI	8:2	627:157
	B11	n-C10/QUI	7:3	548:236
	B12	n-C10/QUI	5:5	392:392
	B13	n-C10/2-Naph	8:2	627:157
	B14	n-C10/2-Naph	7:3	548:236
	B15	n-C10/2-Naph	5:5	392:392
Multicomponent	M1	n-C10/n-C20/c-C10/BuB	4:2:1:1	392:196:98:98
	M2	n-C10/n-C20/QUI/2-Naph	4:2:1:1	392:196:98:98
	M3	n-C10/n-C20/c-C10/BuB/QUI/2-Naph	6:2:1:1:1:1	392:130:65:65:66:66

Note: The total number of oil molecules was fixed at 784 for all binary and multicomponent systems. For system M3, the listed ratio represents the nominal composition, whereas the actual molecular numbers were adjusted to integers while maintaining a total of 784 oil molecules.

**Table 4:** Validation of the simulated densities of CO_2_ (at 363.15 K and 15 MPa) and oil components (at 298.15 K and 1 MPa).

Component	Molar Mass (g/mol)	Simulated Density (g/cm^3^)	Reference Density (g/cm^3^)	Relative Deviation (%)
CO_2_	44.01	0.369	0.372	−0.806
n-C6	86.18	0.662	0.626	5.751
n-C10	142.28	0.734	0.730	0.548
n-C15	212.41	0.774	0.766	1.044
n-C20	282.55	0.796	0.789	0.887
C10iso	142.28	0.740	0.726	1.928
c-C10	140.26	0.842	0.816	3.186
BuB	134.22	0.877	0.860	1.977
QUI	129.16	1.097	1.090	0.642
DecA	172.26	0.896	0.901	−0.555
2-Naph	144.16	1.137	1.141	−0.351

Note: For components whose stable phase at 298.15 K is not liquid, the density comparison is provided only as a reference for condensed-phase consistency and is not interpreted as liquid-phase validation.

## Data Availability

The original contributions presented in this study are included in the article. Further inquiries can be directed to the corresponding author.

## Supplementary Materials

The following supporting information can be downloaded at: https://www.mdpi.com/article/10.3390/molecules31183140/s1, Figure S1: Sensitivity of the calculated extraction characteristics to the spherical counting-boundary radius for the ten single-component oil–CO_2_ systems; Figure S2: Intermolecular center of mass radial distribution functions of 2-Naph within central spherical regions of 15, 20, and 25 Å in the single component 2-Naph–CO_2_ system; Figure S3: Time evolution of mass-transfer characteristics, interaction energy, and system pressure for the ten single-component oil–CO_2_ systems during the 1 ns simulations; Figure S4: Mean-square displacement (MSD) curves of the two oil components in the five binary oil–CO_2_ systems at different compositions; Table S1: Extraction ratios obtained from three independent simulations of the single-component oil–CO_2_ systems, together with the corresponding averages and standard deviations; Table S2: Pearson and Spearman correlations of the single component extraction ratio with imageand image, calculated for the complete component set and after exclusion of 2-Naph; Table S3: Extraction ratios obtained from three independent simulations of the binary oil–CO_2_ systems, together with the corresponding averages and standard deviations; Table S4: Boundary radii determined from the midpoint of the total oil mass density profile for the binary and multicomponent oil–CO_2_ systems in three independent simulations, together with the corresponding averages and standard deviations; Table S5: Extraction ratios obtained from three independent simulations of the multicomponent oil–CO_2_ systems, together with the corresponding averages and standard deviations; Table S6: Lennard–Jones parameters and partial charges used for all molecular species; Table S7: Bond parameters used in the molecular models; Table S8: Angle parameters used in the molecular models; Table S9: Dihedral parameters used in the molecular models.
