# Supplementary material for: Molecular Dynamics Study of CO2-Induced Transfer of Crude Oil Components: Roles of Molecular Structure, Cohesion, and Mixture Composition
Source: Molecules. 2026 Sep 8;31(18):3140. doi: 10.3390/molecules31183140 (PMC13609370; doi:10.3390/molecules31183140)
Supplement: Supplementary file 1 [file molecules-31-03140-s001.zip › molecules-4484736-supplementary.pdf]

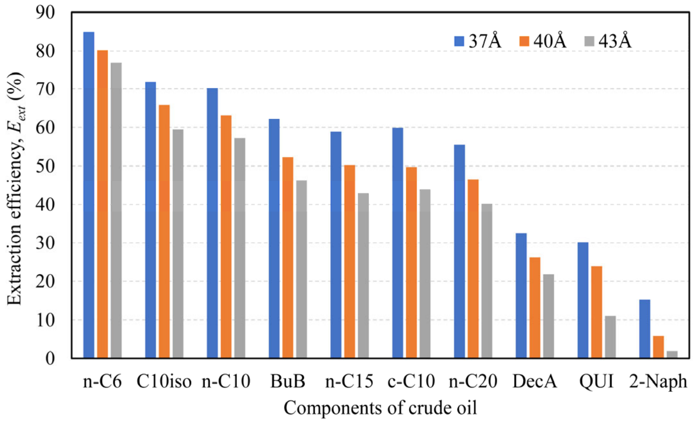

Figure S1. Sensitivity of the calculated extraction characteristics to the spherical counting-boundary radius for the ten single-component oil–CO_2 systems. The numbers of extracted oil molecules were calculated using boundary radii of 37, 40, and 43 Å, with the instantaneous center of mass of all oil molecules used as the center of the counting sphere. Changing the boundary radius affected the absolute extraction counts, whereas the overall component-dependent trend and relative extraction characteristics were preserved.

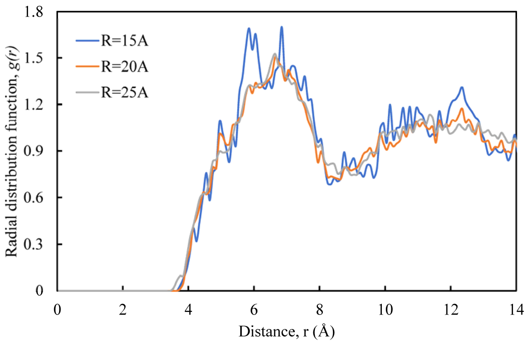

Figure S2. Intermolecular center of mass radial distribution functions of 2-Naph within central spherical regions of 15, 20, and 25 Å in the single component 2-Naph–CO_2 system. The RDFs were averaged over the 800–1000 ps interval and normalized using the analytical pair distance distribution for a finite spherical region.

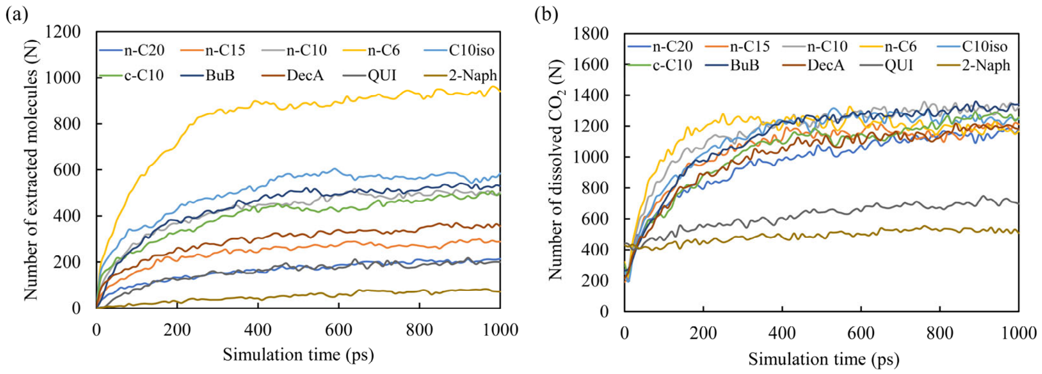

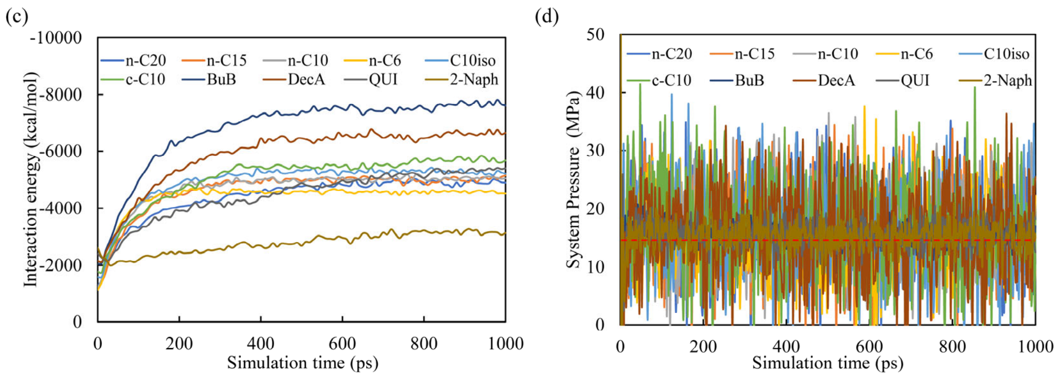

Figure S3. Time evolution of mass-transfer characteristics, interaction energy, and system pressure for the ten single-component oil–CO_2 systems during the 1 ns simulations: (a) number of extracted oil molecules; (b) number of CO_2 molecules dissolved in the oil-rich domain; (c) oil–CO_2 interaction energy; and (d) system pressure. The dashed line in (d) indicates the nominal pressure of 15 MPa. The mean pressures calculated over the statistical sampling interval of 800–1000 ps were 15.76, 15.76, 15.46, 16.09, 15.41, 15.38, 15.43, 15.50, 15.72, and 15.37 MPa for n-C20, n-C15, n-C10, n-C6, C10iso, c-C10, BuB, DecA, QUI, and 2-Naph, respectively.

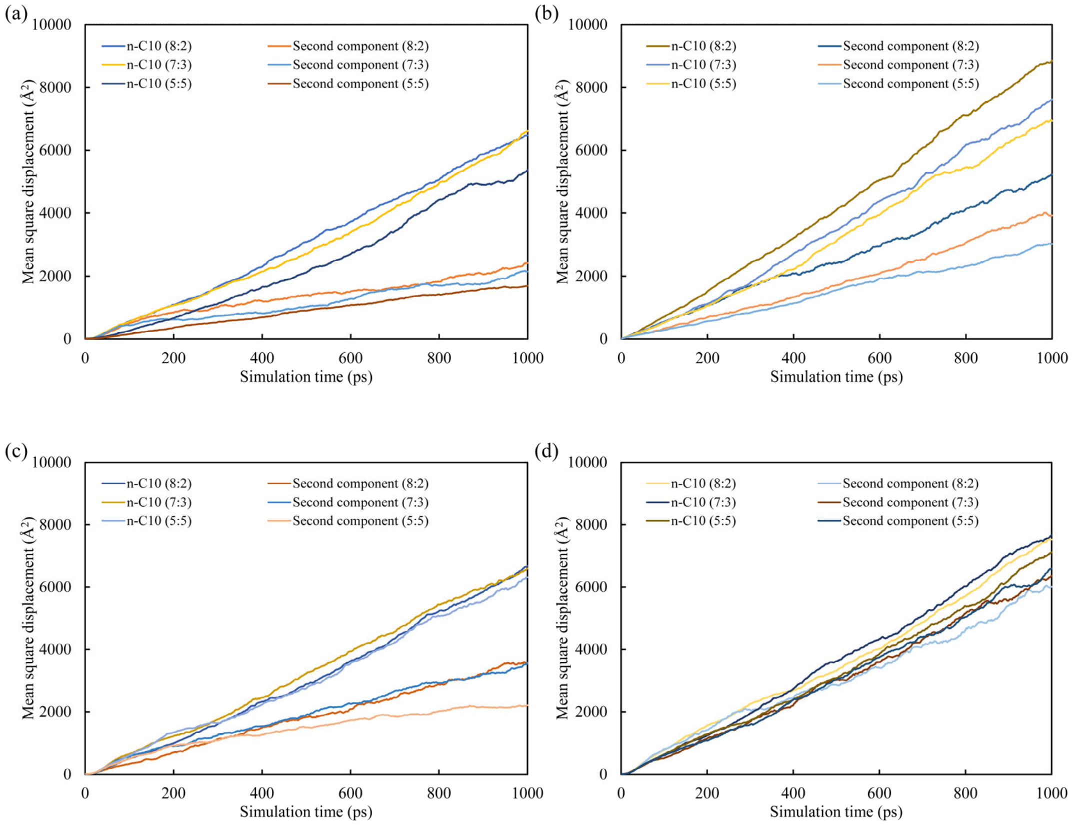

(e)

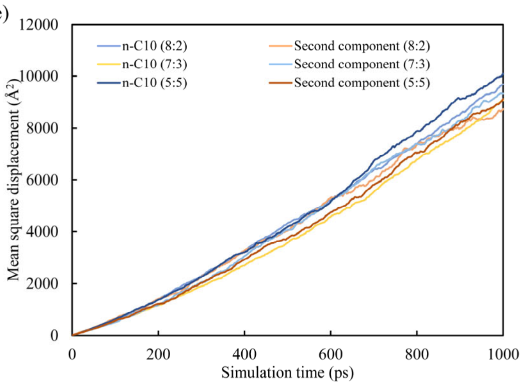

Figure S4. Mean-square displacement (MSD) curves of the two oil components in the five binary oil–CO_2 systems at different compositions: (a) n-C10/2-Naph, (b) n-C10/DecA, (c) n-C10/QUI, (d) n-C10/BuB, and (e) n-C10/C10iso. For each binary system, the MSD curves of both oil components are shown at n-C10:second-component molecular ratios of 8:2, 7:3, and 5:5. The fitting intervals used to calculate the diffusion coefficients were selected from the approximately linear portions of the MSD curves within the 800–1000 ps simulation interval.

Table S1. Extraction ratios obtained from three independent simulations of the single-component oil–CO_2 systems, together with the corresponding averages and standard deviations.

**Components**	**Extraction ratio (%)**			**Average (%)**	**SD (%)**
	**Run1**	**Run2**	**Run3**		
n-C6	80.19	81.56	80.35	80.70	0.75
C10iso	65.88	65.02	63.54	64.82	1.18
n-C10	63.17	63.88	66.24	64.43	1.61
BuB	52.26	53.19	52.74	52.73	0.47
n-C15	50.22	50.74	48.17	49.71	1.36
c-C10	49.75	49.35	48.65	49.25	0.55
n-C20	46.60	51.32	47.98	48.63	2.43
DecA	26.17	26.27	28.15	26.87	1.11
QUI	23.97	24.87	23.65	24.16	0.63
2-Naph	5.82	5.86	5.86	5.85	0.03

Table S2. Pearson and Spearman correlations of the single component extraction ratio with $\Delta\delta_{\mathrm{MD}}$ and R_{comp}, calculated for the complete component set and after exclusion of 2-Naph.

Relationship	Coefficient	All components, n=10	Excluding 2-Naph, n=9	Change in magnitude
E_{ext} vs. $\Delta\delta_{MD}$	Pearson r	−0.933	−0.882	−0.050

Relationship	Coefficient	All components, n=10	Excluding 2-Naph, n=9	Change in magnitude
	Spearman ρ	−0.915	−0.883	−0.032
E_{ext} vs. R_{comp}	Pearson r	0.951	0.948	−0.003
	Spearman ρ	1.000	1.000	0.000

Table S3. Extraction ratios obtained from three independent simulations of the binary oil–CO_2 systems, together with the corresponding averages and standard deviations:

(a) n-C10 component

**Systems**	**n-C10 proportion (%)**	**Extraction ratio (%)**			**Average (%)**	**SD (%)**
		**Run1**	**Run2**	**Run3**		
n-C10+C10iso	80	63.07	63.46	63.17	63.23	0.20
	70	72.15	71.88	70.65	71.56	0.80
	50	73.23	73.37	74.01	73.54	0.42
n-C10+BuB	80	64.92	63.17	63.76	63.95	0.89
	70	64.75	63.99	65.41	64.72	0.71
	50	71.86	72.86	71.89	72.20	0.57
n-C10+DecA	80	73.05	74.22	74.87	74.05	0.92
	70	67.76	65.91	67.62	67.10	1.03
	50	61.12	61.47	60.56	61.05	0.46
n-C10+QUI	80	79.72	80.74	81.66	80.71	0.97
	70	79.06	78.89	78.11	78.69	0.51
	50	72.15	70.99	71.56	71.57	0.58
n-C10+2-Naph	80	71.19	73.01	72.15	72.12	0.91
	70	65.09	66.77	66.10	65.99	0.85
	50	66.98	65.17	64.48	65.54	1.29

(b) Second component

**Systems**	**Second component proportion (%)**	**Extraction ratio (%)**			**Average (%)**	**SD (%)**
		**Run1**	**Run2**	**Run3**		
n-C10+C10iso	20	63	62.71	64.37	63.36	0.89
	30	70.3	70.31	71.89	70.83	0.92
	50	72.69	72.98	73.66	73.11	0.50
n-C10+BuB	20	61.3	62.89	62.99	62.39	0.95

	30	64.87	63.14	65.77	64.59	1.34
	50	68.24	69.99	69.13	69.12	0.88
n-C10+DecA	20	73.58	76.11	75.44	75.04	1.31
	30	54.64	56.17	55.79	55.53	0.80
	50	48.6	47.66	49.71	48.66	1.03
n-C10+QUI	20	66.09	65.55	65.97	65.87	0.28
	30	58.37	58.19	59.33	58.63	0.61
	50	27.02	29.11	30.14	28.76	1.59
n-C10+2-Naph	20	46.38	47.51	46.77	46.89	0.57
	30	27.08	27.09	28.14	27.44	0.61
	50	21.48	20.11	20.97	20.85	0.69

Table S4. Boundary radii determined from the midpoint of the total oil mass density profile for the binary and multicomponent oil–CO_2 systems in three independent simulations, together with the corresponding averages and standard deviations.

Systems	n-C10 proportion (%)	Boundary radii (Å)		
		Run1	Run2	Run3
n-C10+C10iso	8:2	43.15	41.99	42.38
	7:3	36.07	36.64	37.71
	5:5	38.02	38.18	38.14
n-C10+BuB	8:2	38.36	37.69	37.19
	7:3	34.80	35.17	35.55
	5:5	38.75	38.60	37.81
n-C10+DecA	8:2	36.44	37.16	36.45
	7:3	37.83	38.82	38.17
	5:5	39.06	39.48	39.11
n-C10+QUI	8:2	29.16	29.43	29.77
	7:3	28.77	28.67	29.06
	5:5	31.88	32.01	31.14
n-C10+2-Naph	8:2	32.86	32.40	31.37
	7:3	34.04	33.88	33.97
	5:5	33.47	34.17	33.44
M1	/	33.67	34.10	33.71

M2	/	40.35	40.75	40.00
M3	/	37.54	38.14	38.62

Table S5. Extraction ratios obtained from three independent simulations of the multicomponent oil–CO_2 systems, together with the corresponding averages and standard deviations.

Systems	Component	Extraction ratio (%)			Average (%)	SD (%)
		Run1	Run2	Run3		
M1	n-C10	75.34	75.57	74.92	75.28	0.33
	n-C20	75.59	75.81	76.71	76.04	0.59
	c-C10	75.9	76.88	76.43	76.40	0.49
	BuB	74.88	75.35	73.91	74.71	0.73
M2	n-C10	60.00	61.10	60.48	60.53	0.55
	n-C20	41.47	40.04	42.33	41.28	1.16
	QUI	29.40	30.20	29.66	29.75	0.41
	2-Naph	33.38	33.06	32.81	33.08	0.29
M3	n-C10	66.53	67.16	67.46	67.05	0.47
	n-C20	52.45	52.41	51.12	51.99	0.76
	c-C10	58.32	58.14	59.17	58.54	0.55
	BuB	39.41	40.34	41.06	40.27	0.83
	QUI	46.08	45.80	46.71	46.20	0.47
	2-Naph	33.77	31.61	32.01	32.46	1.15

Table S6. Lennard–Jones parameters and partial charges used for all molecular species.

Component	Site/Atom type	ε (kcal mol^{-1})	σ (Å)	q (e)
CO_2	O	0.15699	3.05	−0.350
	C	0.0537	2.8	0.7
n-C6/n-C10/n-C15/n-C20	CH_3	0.194746	3.75	0
	CH_2	0.091411	3.95	0
C10iso	CH_3	0.194746	3.75	0
	CH_2	0.091411	3.95	0
	CH	0.019872	4.68	0
c-C10	CH_2	0.101347	3.98	0
BuB	Csub	0.09539	3.74	0
	CH	0.09539	3.74	0
	Mcenter	0	0	2.42

	Mπ	0	0	−1.210
	CH_2	0.09141	3.95	0
	CH_3	0.19475	3.75	0
DecA	CH_3	0.194746	3.75	0
	CH_2	0.091411	3.95	0
	CH_2COOH	0.091411	3.95	0.12
	CACID	0.081475	3.9	0.42
	OCO	0.156989	3.05	−0.450
	OOH	0.18481	3.02	−0.460
	HO	0	0	0.37
QUI	N1	0.113271	3.2	−0.374
	C2	0.061007	3.6	0.121
	C3	0.061007	3.6	−0.107
	C4	0.061007	3.6	−0.032
	C5	0.061007	3.6	−0.050
	C6	0.061007	3.6	−0.077
	C7	0.061007	3.6	−0.067
	C8	0.061007	3.6	−0.158
	C8A	0.109296	3.2	0.089
	C4A	0.109296	3.2	−0.010
	H2	0.050574	2.36	0.081
	H3	0.050574	2.36	0.108
	H4	0.050574	2.36	0.1
	H5	0.050574	2.36	0.095
	H6	0.050574	2.36	0.101
	H7	0.050574	2.36	0.098
	H8	0.050574	2.36	0.082
2-Naph	C1	0.061007	3.6	0.092
	C2	0.061007	3.6	−0.132
	C3	0.061007	3.6	−0.063
	C4	0.061007	3.6	−0.105
	C5	0.061007	3.6	−0.082
	C6	0.061007	3.6	−0.076
	C7	0.061007	3.6	−0.085
	C8	0.061007	3.6	−0.071
	C8A	0.109296	3.2	−0.050
	C4A	0.109296	3.2	−0.018

O11	0.23449	3.04	−0.486
HO	0.023846	0.5	0.435
H2	0.050574	2.36	0.09
H3	0.050574	2.36	0.1
H4	0.050574	2.36	0.088
H5	0.050574	2.36	0.088
H6	0.050574	2.36	0.095
H7	0.050574	2.36	0.098
H8	0.050574	2.36	0.082

Table S7. Bond parameters used in the molecular models.

Component	Bond type	K_b	r_0 (Å)
CO_2	C–O	2019.853	1.16
n-C6/n-C10/n-C15/n-C20	C–C	1000	1.54
C10iso	C–C	1000	1.54
c-C10	C–C	1000	1.54
BuB	Aromatic C–C	1000	1.4
	Mcenter–Mπ	1000	0.785
	Mcenter–C1	1000	1.4
	Csub–CH_2 / alkyl C–C	1000	1.54
DecA	Alkyl C–C	1000	1.54
	CH_2–CACID	1000	1.52
	CACID–OCO	1000	1.214
	CACID–OOH	1000	1.364
	OOH–HO	1000	0.97
QUI	N1–C2	2000	1.31
	C2–C3	2000	1.41
	C3–C4	2000	1.37
	C4–C4A	2000	1.41
	C4A–C5	2000	1.41
	C5–C6	2000	1.37
	C6–C7	2000	1.41
	C7–C8	2000	1.37
	C8–C8A	2000	1.41
	C8A–N1	2000	1.36
	C8A–C4A	2000	1.41

	C2–H2 / C3–H3 / C4–H4 / C5–H5 / C6–H6 / C7–H7 / C8–H8	2000	1.08
2-Naph	C1–C2	2000	1.37
	C2–C3	2000	1.41
	C3–C4	2000	1.37
	C4–C4A	2000	1.41
	C4A–C5	2000	1.41
	C5–C6	2000	1.37
	C6–C7	2000	1.41
	C7–C8	2000	1.37
	C8–C8A	2000	1.41
	C8A–C1	2000	1.41
	C8A–C4A	2000	1.42
	C1–O11	2000	1.36
	O11–HO	2000	0.95
	C2–H2 / C3–H3 / C4–H4 / C5–H5 / C6–H6 / C7–H7 / C8–H8	2000	1.08

Table S8. Angle parameters used in the molecular models.

Component	Angle type	K_θ	θ_0 (°)
CO_2	O–C–O	108.1098	180
n-C6/n-C10/n-C15/n-C20	C–C–C	124.2003	114
C10iso	CHx–CH_2–CHy	62.1001331	114
	CHx–CH–CHy	62.1001331	112
c-C10	C–C–C	62.1001	114
BuB	Aromatic C–C–C	500	120
	C1–Mcenter–C4	500	180
	Mπ–Mcenter–Mπ	500	180
	C1–Mcenter–Mπ	500	90
	C–CH_2–C / alkyl CH_2-centered	62.1	114
DecA	Alkyl C–C–C	124.2002662	114
	CH_2–CACID–OCO	80.08433162	126
	CH_2–CACID–OOH	70.14831033	111
	OCO–CACID–OOH	80.08433162	123
	CACID–OOH–HO	34.97479495	107
QUI	N1–C2–C3	2000	124.13

	C2–C3–C4	2000	118.71
	C3–C4–C4A	2000	119.31
	C4–C4A–C8A	2000	117.52
	C4A–C8A–N1	2000	122.38
	C8A–N1–C2	2000	117.94
	C5–C6–C7	2000	120.23
	C6–C7–C8	2000	120.65
	C7–C8–C8A	2000	120.4
	C8–C8A–C4A	2000	118.97
	C8A–C4A–C5	2000	119.25
	C4A–C5–C6	2000	120.5
	H2–C2–C3	2000	119.55
	H3–C3–C4	2000	121.52
	H4–C4–C4A	2000	119.45
	H5–C5–C6	2000	120.64
	H6–C6–C7	2000	119.61
	H7–C7–C8	2000	119.92
	H8–C8–C8A	2000	117.73
2-Naph	C1–C2–C3	2000	120.24
	C2–C3–C4	2000	120.75
	C3–C4–C4A	2000	120.21
	C4–C4A–C8A	2000	119.68
	C4A–C8A–C1	2000	118.32
	C8A–C1–C2	2000	120.79
	C5–C6–C7	2000	120.34
	C6–C7–C8	2000	120.24
	C7–C8–C8A	2000	120.49
	C8–C8A–C4A	2000	119.6
	C8A–C4A–C5	2000	118.21
	C4A–C5–C6	2000	121.11
	O11–C1–C2	2000	122.69
	H2–C2–C3	2000	119.94
	H3–C3–C4	2000	120.21
	H4–C4–C4A	2000	119.08
	H5–C5–C6	2000	120.42
	H6–C6–C7	2000	119.66
	H7–C7–C8	2000	120.03

H8–C8–C8A	2000	118.81
HO–O11–C1	2000	109.3

Table S9. Dihedral parameters used in the molecular models.

Component	Dihedral type	K_1	K_2	K_3	K_4
n-C6/n-C10/n-C15/n-C20	Alkane C–C–C–C	0.71	-0.14	1.57	0.00
C10iso	CH_x–CH_2–CH–CH_y	1.70	-0.44	1.75	0.00
	CH_x–CH_2–CH_2–CH_y	1.41	-0.27	3.15	0.00
c-C10	Ring C–C–C–C	1.41	-0.27	3.15	0.00
BuB	Aromatic planarity	0.00	50.00	0.00	0.00
	Aryl–alkyl	0.00	-0.52	0.00	0.00
	Alkyl C–C–C–C	1.41	-0.27	3.14	0.00
DecA	Alkane	1.41	-0.27	3.15	0.00
	CH_x–CH_2–CACID–OCO	-2.50	3.10	0.00	0.00
	CH_x–CH_2–CACID–OOH	1.41	-0.27	3.15	0.00
	OCO–CACID–OOH–HO	-2.50	3.10	0.00	0.00
	CH_2COOH–CACID–OOH–HO	2.50	3.10	0.00	0.00
CO_2	—	—	—	—	—
QUI	—	—	—	—	—
2-Naph	—	—	—	—	—
